# Non-Invasive geophysical imaging reveals Ptolemaic tombs at Al-Dyabat for global archaeological applications

**DOI:** 10.1038/s41598-025-23113-9

**Published:** 2025-11-04

**Authors:** Abdelbaset M. Abudeif, Mohammed A. Mohammed, Hossameldeen A. Gaber

**Affiliations:** Faculty of science, Geology department, Sohag University, Sohag, Egypt

**Keywords:** Geophysical survey, Magnetic method, GPR, Ptolemaic tombs, Al-Dyabat, Akhmim, Egypt, Planetary science, Solid Earth sciences

## Abstract

This study presents a geophysical investigation aimed at identifying Ptolemaic Period tombs and associated archaeological structures at Al-Dyabat archaeological hill, near Akhmim City, Egypt. The site gained international attention following the 2018 discovery of the tomb of the Priest Tutu. Combined ground magnetic surveying and ground-penetrating radar (GPR) were used at the promising selected site. Integrated analysis revealed subsurface anomalies interpreted as tomb chambers, mudbrick walls, and possible limestone coffins, located at depths between 0.2 and 3 m. The combination of magnetic and GPR data significantly enhanced the detection capabilities and structural resolution, demonstrating the effectiveness of multimodal geophysics in archaeological contexts. Beyond its regional relevance, this work offers a transferable model for non-invasive archaeological prospection in sensitive heritage zones worldwide. It underscores the value of geophysical integration in cultural heritage preservation, offering insights into best practices for sustainable and non-destructive archaeological exploration.

## Introduction

The exploration of new archaeological sites using integrated geophysical methods has become increasingly vital, as such investigations not only deepen our understanding of past civilizations but also have significant implications for cultural heritage management and economic development. Geophysical discoveries offer critical insights into ancient societies while contributing to the national economy through enhanced tourism and heritage conservation. Archaeological sites, as cultural landmarks, attract millions of visitors annually, generating substantial revenue and creating diverse employment opportunities.

Among the available techniques, near-surface geophysical methods have proven to be highly effective for investigating buried archaeological features non-invasively, avoiding the physical disturbance often caused by excavation^1^. These methods are now regarded by archaeologists as essential modern tools that allow for the detection and mapping of subsurface structures beyond the reach of conventional field techniques^2^. Many authors used integration of geophysical methods to detect many archaeological features ^2–18^.

Egypt is home to numerous significant archaeological cities, among which Akhmim, located in Upper Egypt, holds particular importance. The city encompasses extensive necropolises that date back to the 6th Dynasty (2325–2150 BCE) and extend through to the late Coptic period. These burial grounds have yielded a wealth of archaeological artifacts, including textiles and objects from various historical eras.

In archaeological geophysics, magnetic surveying is often the first method employed due to its high efficiency in detecting shallow subsurface features, especially those constructed from mudbrick and fired brick. Its effectiveness has been well documented in numerous studies across Egypt^12,16,17,19–29^. In addition to magnetometry, ground-penetrating radar (GPR) has become increasingly valuable in archaeological investigations. GPR is capable of detecting subsurface features composed of a wide range of materials with high accuracy, generating detailed two- and three-dimensional images of buried structures at shallow depths, from just a few centimeters to several meters^30^. Its successful application in archaeology has been demonstrated in several studies (e.g. ^31-37^).

The primary objective of this study is to identify and delineate potential archaeological features buried at shallow depths within the Al-Dyabat archaeological hill in Akhmim City, Egypt. A key focus is the detection of Ptolemaic tombs, which are known to exhibit a variety of architectural styles, including rock-cut chambers and brick-lined graves. The study area is considered a largely unexplored site with minimal prior archaeological documentation. To achieve this objective, an integrated geophysical approach was employed. Ground magnetic surveying and ground-penetrating radar (GPR) were selected due to their proven effectiveness in previous archaeological investigations across Egypt and internationally. The results of this study are expected to support archaeologists in reconstructing the subsurface layout of the site and in locating additional tombs or structures that are not visible at the surface.

Beyond its scientific significance, this study carries broader implications for heritage tourism and national economic development. As Egypt continues to invest in the sustainable promotion of its cultural assets, the integration of non-invasive geophysical methods in archaeological site exploration represents a strategic tool for discovering and preserving hidden heritage without destructive excavation. The identification of new tombs and architectural remains at Al-Dyabat, a site with growing historical relevance, can contribute to expanding the archaeological tourism map in Upper Egypt. Such discoveries are likely to attract both national and international visitors, stimulate local economies, and enhance Egypt’s global profile as a leading destination for cultural tourism. By offering a replicable model for heritage prospection, this research supports long-term strategies for increasing tourism revenue while safeguarding archaeological resources for future generations.

## Location map and description of the study area

**Fig. 1:**
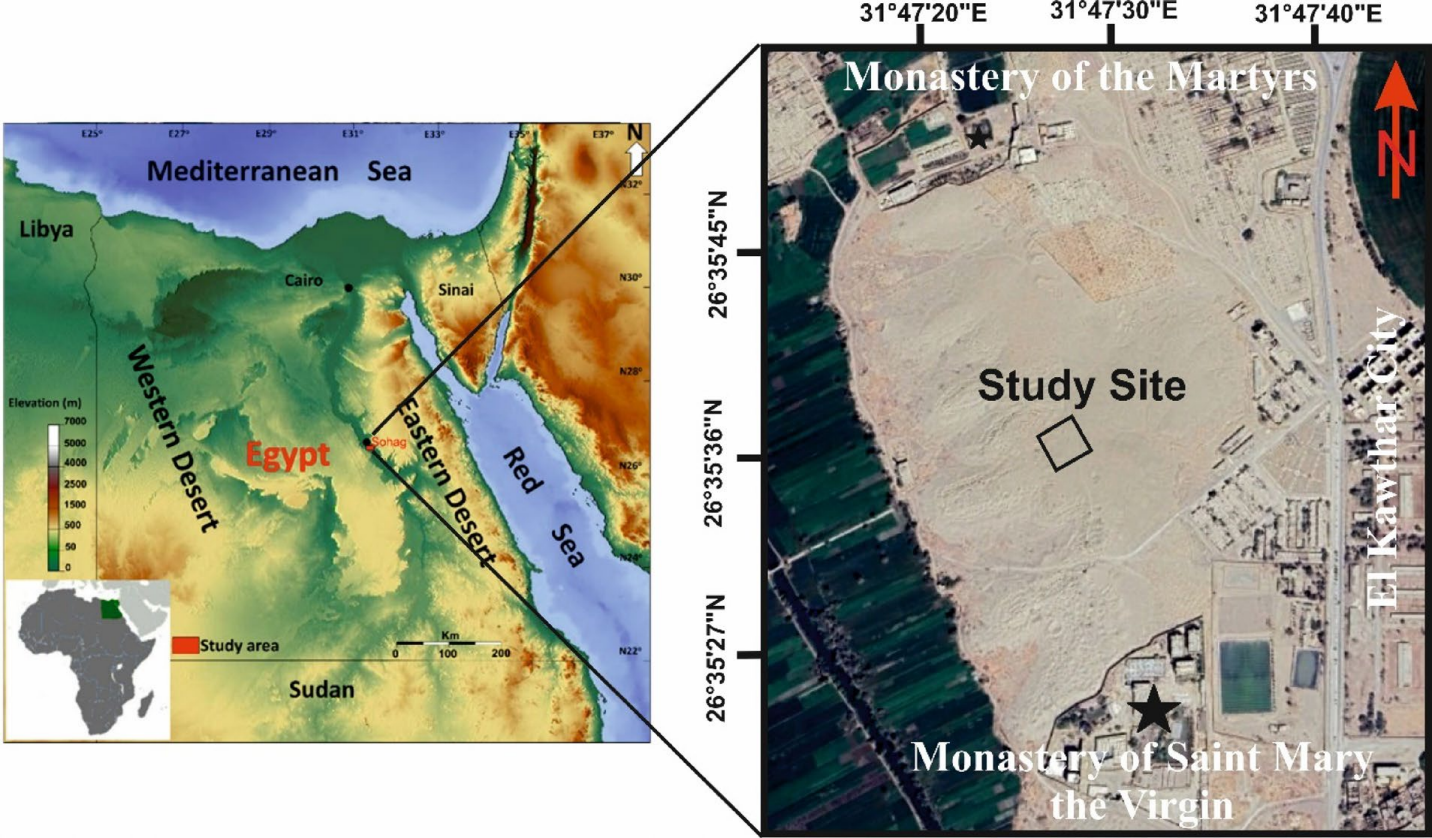
Location map of the study area. The left panel shows the regional context of the Al-Dyabat archaeological hill near Akhmim City, Upper Egypt. The right panel presents a high-resolution satellite image of the site, indicating the positions of the investigated site in the middle part between the Monastery of Saint Mary the Virgin in the South and the Monastery of the Martyrs in the North. The map highlights the proximity of the surveyed zones to significant modern landmarks and archaeological features.

The study area is part of the Al-Dyabat archaeological hill, situated approximately 5 km east of Akhmim City in the Sohag Governorate, Egypt. Geographically, it extends between latitudes 26°35’27’’N and 26°35’45’’N, and longitudes 31°47’20’’E and 31°47’40’’E (Fig. 1). The hill is bordered to the west by agricultural land and to the east by El Kawther City. To the north lies the Monastery of the Martyrs, while the Monastery of Saint Mary the Virgin is located to the south.

**Fig. 2:**
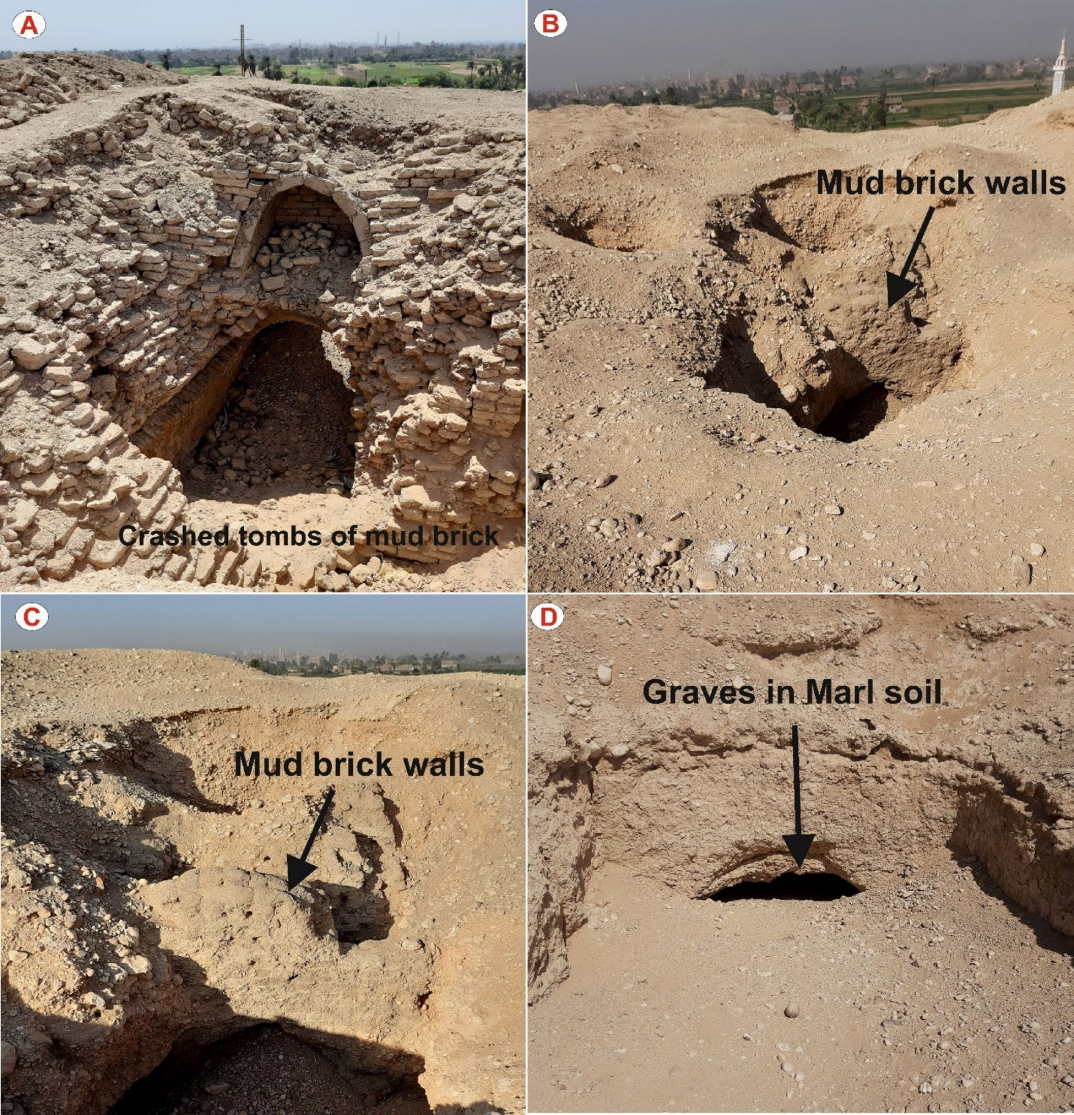
Photographic documentation of exposed archaeological features at Al-Dyabat archaeological hill, Akhmim City, Egypt. (**A**): Collapsed tomb constructed of mud bricks. (**B** and **C**): Exposed mudbrick walls of burial structures revealed through partial erosion. (**D**): Rock-cut grave carved into marl soil, showing an arched entrance typical of shallow Ptolemaic burials. These surface exposures provide critical insights into the burial architecture and construction materials, supporting geophysical interpretation and site characterization.

Since February 2018, the site has been officially recognized by the Egyptian Ministry of Tourism and Antiquities as an archaeological area, following the significant discovery of the tomb of the Priest Tutu, which dates back to the Ptolemaic period^38^. The site contains numerous tombs, some of which are hewn directly into layers of clay and marl, while others are constructed from mud brick (Fig. 2). The significance of this location is further emphasized by the fact that it represents the pioneer geophysical investigation conducted on this recently discovered archaeological hill, as no international archaeological missions have yet commenced work in the area.

## Geological setting

**Fig. 3:**
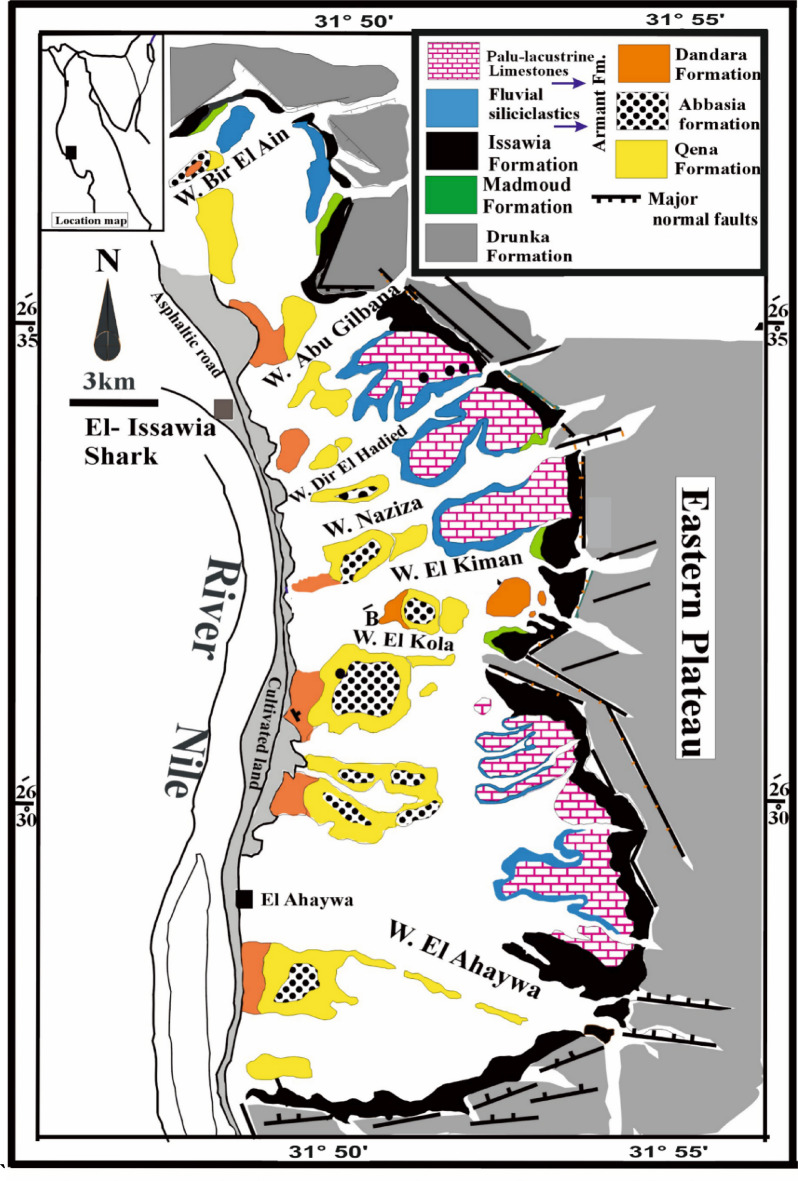
Surface lithostratigraphic map of eastern Sohag, including the study area, showing the distribution of major geological formations and structural features. The map highlights key units such as the Issawia, Madmoud, Drunka, and Dandara formations, as well as palaeo-lacustrine limestones, fluvial siliciclastics, and major normal faults, modified after^40^.

The study area is situated in the eastern sector of the Nile Valley within Akhmim City, Sohag Governorate, Egypt. Numerous geological investigations have been carried out to examine the lithostratigraphy of the sedimentary sequences in the Sohag region^39–44^. According to ^40^, the geological formations in this region primarily consist of sedimentary successions ranging in age from the Lower Eocene to the Recent (Fig. 3). The principal geological formations present within the study area are summarized in Table 1. Structurally, the area is characterized by significant tectonic activity, including faulting, folding, and the presence of major joint systems^40^.

**Table 1:** Major geological formations in East Sohag area including the study site.

Formation	Description	Age	References
Recent wadi deposits	broken Eocene carbonate and reworked material from the pre–existing sediments.	Holocene (Recent)	^40^
Alluvial deposits (Nile floodplain)	Clays and silts with intercalations of sandstone		^41^
Dandara	massive siltstone and fine-grained sandstone, grades upward into gypsiferous claystone with lenticular bodies of hard sandstone	Pleistocene	^44^
Abbasia	Conglomerates that are composed of igneous clasts, quartzite, chert and silicified limestone embedded in red sandy matrix.		^45^
Qena	A thick succession of cross bedded, friable to semi-consolidated fluviatile sands and gravels.		^43^
Armant	fine grained clastic beds alternating with bedded travertine carbonates.		^45^
Issawia	Clastic facies of gravels, cobbles and pebbles of limestone and flint in a calcareous matrix and carbonate facies of travertine, limestone and Tufa.		^46^
Madmoud	The upper section consists of brown to yellow sandstones, siltstones and claystones. The lower section is a thick succession of brown and grey clays intercalated with thin beds of silts and fine sands.	Pliocene	^45^
Drunka	Snow white to grayish white color of medium- to thick-bedded limestone that contains chert bands and bioturbated with Echinoderms, Mollusca, large Foraminifera, and Calcareous Algae in some horizons.	Lower Eocene	^47^
Thebes	Massive to layered limestone with flint nodules, as well as marls rich in Nummulites, Assilines and Operculines		^47^

## Archaeological background

Akhmim, historically known as Ipu or Khent-min, is one of the oldest cities in Upper Egypt, with a rich archaeological legacy extending back to the Pharaonic era, approximately six thousand years ago^48^. The city served as a major religious and administrative center, dedicated to the fertility god Min, who formed a triad with the deities Isis and Horus^49^. Akhmim was the capital of the Ninth Nome of Upper Egypt^50^. Numerous archaeological excavations have revealed a wealth of artifacts, such as sculptures, stelae, and temple ruins, underscoring its importance during the Old Kingdom and subsequent periods. However, the extent of visible monuments in Akhmim today is limited due to the modern city being built directly atop the ancient one. Notable discoveries include a colossal statue of Queen Meritamun, daughter of Ramses II, which further affirms Akhmim’s prominence during the New Kingdom^49^.

The archaeological record of Akhmim offers invaluable insights into ancient Egyptian religious practices, artistic traditions, and administrative systems. During the Greco-Roman period, the city, then known as Panopolis, emerged as a renowned religious and artistic hub. It became especially notable for its textile production, particularly fine linen, which was exported throughout the Roman Empire. Archaeological finds from this era include elaborately woven textiles and early Coptic manuscripts, attesting to the city’s economic prosperity and cultural vitality^51^. Additionally, Akhmim was known for its metalworking and shipbuilding industries^50^.

In the Coptic period, Akhmim gained prominence as a significant Christian center, marked by the construction of numerous churches and monasteries. The architectural remains, religious carvings, and Coptic art from this period reflect the city’s role in the spread of Christianity in Egypt.

Akhmim’s necropolis, located on the eastern bank of the Nile, includes the tombs of elite individuals, further highlighting its role as a spiritual and political center. Among the most prominent burial sites is the Hawawish necropolis, which dates primarily to the Old Kingdom (2686–2181 BCE) and the First Intermediate Period (2181–2055 BCE)^52^. These rock-cut tombs, hewn into limestone cliffs, served as the final resting places for high-ranking officials, priests, and local elites. The tombs of Hawawish are noted for their distinctive artistic styles, which merge conventional Egyptian motifs with local influences^53^.

**Fig. 4:**
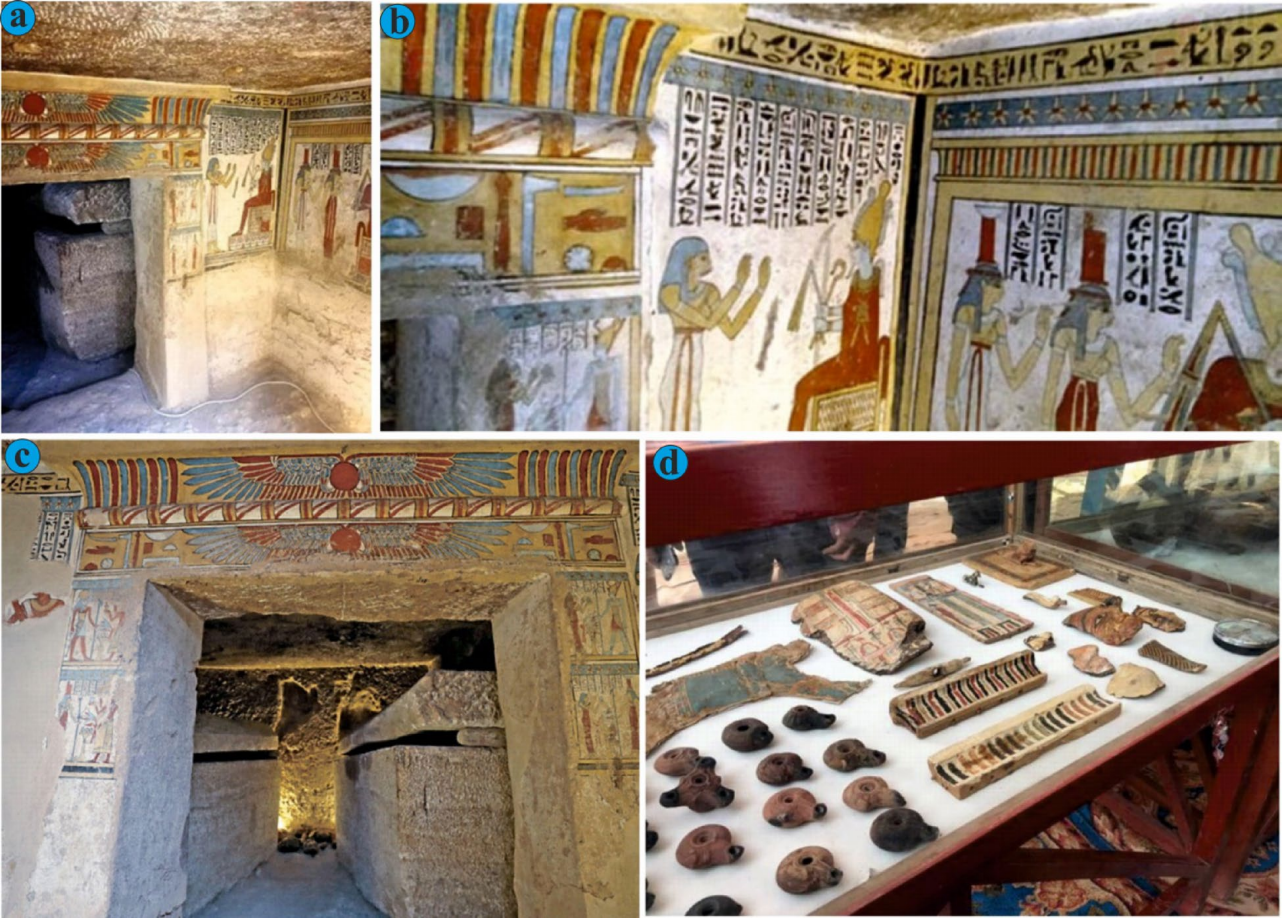
Photographic documentation of an elaborately decorated Priest Tutu tomb in 2018, showcasing funerary iconography, hieroglyphic inscriptions, and burial objects (Photos courtesy of the Egyptian Museum of Antiquities): (**a**) General view of a tomb chamber with walls adorned with colorful hieroglyphic inscriptions and ritual scenes. The texts include invocations to deities such as Osiris and Anubis, requesting offerings and eternal life for the deceased. (**b**) Close-up of a painted funerary scene showing the deceased making offerings to seated deities. Vertical columns of hieroglyphs include traditional offering formulas (ḥtp dỉ nsw) and references to divine names, such as Isis, Nephthys, and Horus. (**c**) Interior passageway crowned with a winged sun disk flanked by uraei, symbolizing protection by the solar deity Ra. Flanking texts contain protective spells and references to the journey through the underworld. (**d**) Display case containing funerary artifacts recovered from the tomb, including painted wooden panels (likely coffin fragments), faience amulets, terracotta oil lamps, personal grooming items such as combs, and inscribed fragments of cartonnage. Images courtesy of the Egyptian Museum of Antiquities.

In February 2018, the Egyptian Ministry of Antiquities announced the discovery of the Tomb of the Priest Tutu at the Al-Dayabat archaeological site near Akhmim, Sohag Governorate. The tomb was uncovered accidentally when a group was apprehended conducting illegal excavations. This tomb, dating back to the Ptolemaic period (305–30 BCE), is considered one of the most significant archaeological finds in recent years. It comprises a central hall with a burial shaft leading to two chambers, the walls of which are adorned with vivid and remarkably well-preserved reliefs and inscriptions (Fig. 4). These decorations provide critical insights into the iconography and religious practices of the Ptolemaic period^38^.

The tomb’s historical importance lies in its representation of a period during which Egypt was under Greek rule yet continued to uphold ancient Egyptian traditions. This cultural blend is evident in both the artistic and architectural elements of the tomb. Religiously, the tomb underscores the enduring role of priests in maintaining ritual practices. Depictions of deities such as Isis, Osiris, and Horus emphasize the continuity of traditional beliefs during this transitional era.

## Materials and methods

An integrated geophysical approach combining ground magnetic surveying (total magnetic intensity and vertical magnetic gradient) and ground-penetrating radar (GPR) was employed at the selected site to enhance the effectiveness of archaeological prospecting. These complementary methods were selected to meet the constraints of limited resources, offering time- and cost-efficient data acquisition.

## Magnetic method

Magnetic surveying has become a cornerstone of archaeogeophysical investigations due to its capability to detect subsurface archaeological features such as tombs, mudbrick structures, entrances, and walls^4^. The magnetic contrast between archaeological features and the surrounding matrix, whether of natural or anthropogenic origin, results in distinct, interpretable anomalies^7^.

**Fig. 5:**
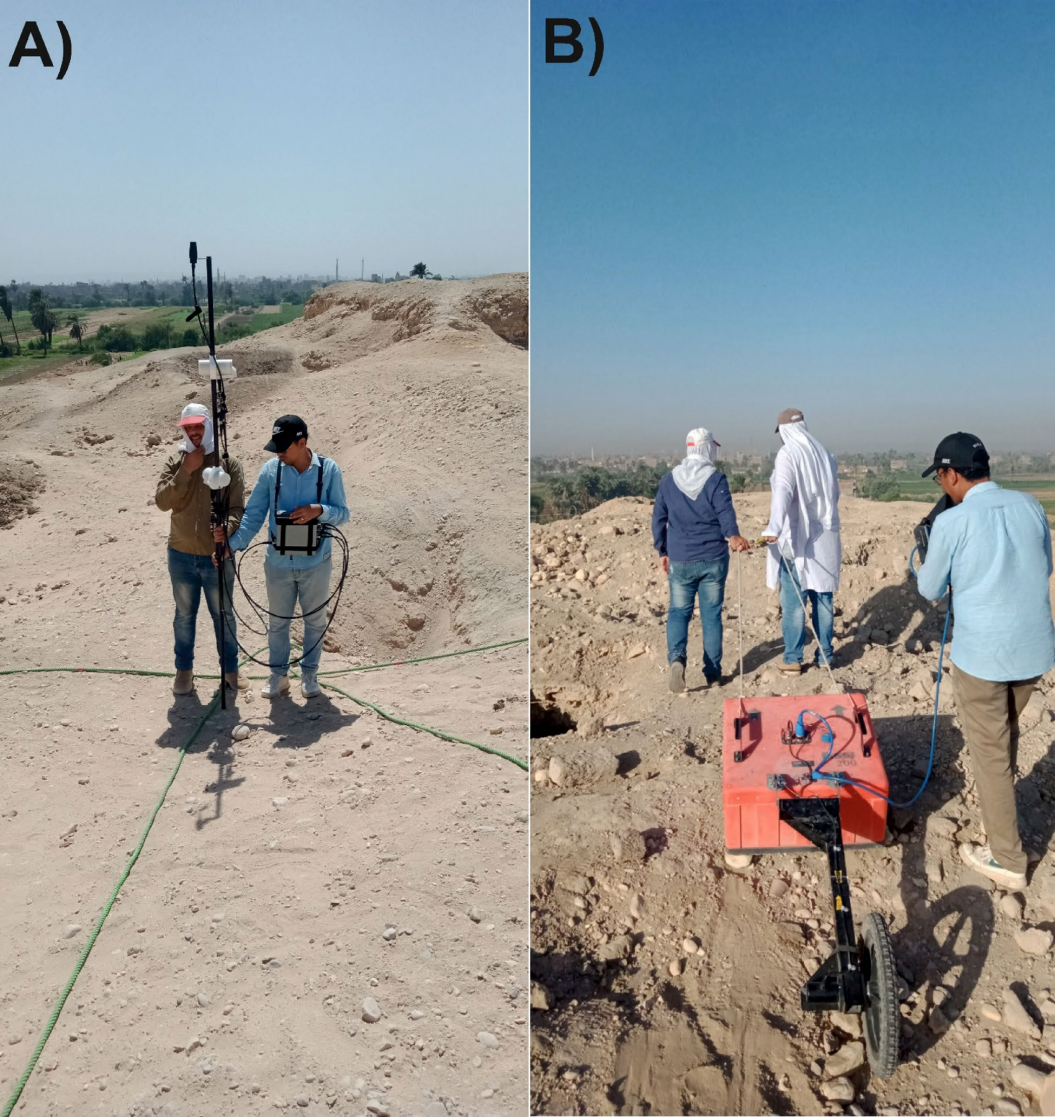
Photographs documenting the geophysical fieldwork conducted over the archaeological hill: (**A**) Magnetic survey using a GEM GSM-19 Overhauser magnetometer equipped with dual sensors for enhanced data resolution. (**B**) Ground Penetrating Radar (GPR) survey using a GSSI SIR 4000 system with a 200 MHz antenna mounted on a survey wheel to ensure consistent data acquisition across uneven terrain.

In this study, the study site was surveyed using a GEM GSM-19 Overhauser magnetometer with dual sensors. The lower sensor was positioned 1.5 m above ground, and the vertical separation between sensors was 0.6 m (Fig. 5a). This system, owned by the Geology Department at Sohag University, has a sensitivity of 0.022 nT and a resolution of 0.01 nT. All measurements were taken under noise-free conditions; metallic objects on the ground surface were cleared in advance to avoid data contamination. The site measured 50 × 50 m (2500 m²) and was surveyed in NW–SE-oriented zigzag transects with 1 m line spacing. The magnetometer operated in continuous mode, logging data every 2 s, yielding 1210 readings.

**Fig. 6:**
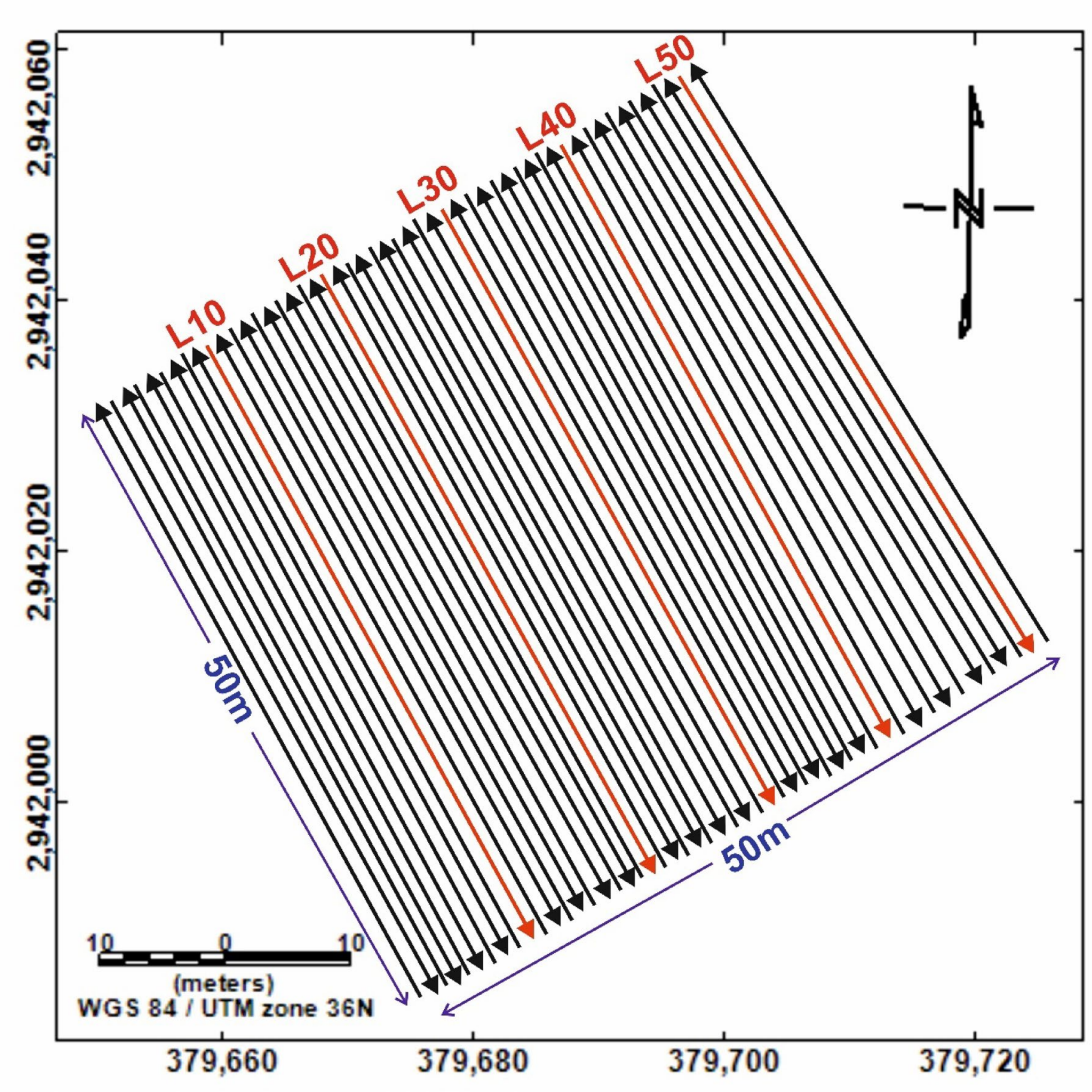
Layout of the magnetic survey configuration at the study site with a 50 m × 50 m coverage. Data were acquired along NW–SE oriented lines at 1-meter spacing in a zigzag pattern. Coordinate system: WGS 84 / UTM zone 36 N.

The magnetic dataset included both total magnetic intensity (TMI) from the bottom sensor and vertical magnetic gradient, calculated as the difference between the two sensors divided by their separation^54^. Figure 6 illustrates the acquisition layout.

Magnetic data processing was conducted using Geosoft Oasis Montag Software ver. 8.4 ^55^, applying the following steps to enhance signal quality and reveal archaeological features:

- Butterworth High-Pass Filter: Applied in the frequency domain with an 8 m cutoff and degree 8, to suppress low-frequency anomalies and highlight shallow features.
- The derivative or gradient approaches improve the clarity of anomalies and bring out shallow features more clearly^56^. Among these derivatives, the vertical derivative map is more sensitive to local impacts than to larger regional effects. Both the first vertical derivative (FVD) and second vertical derivative (SVD) approaches are more sensitive to shallow magnetic sources than total field magnetic data, but they can also amplify noise^57,58^.
- The tilt angle derivatives (TDR) approach is defined as the arctan of the ratio of vertical to horizontal derivatives, and its zero-contour line represents the magnetic susceptibility source’s margins^59^. This filter is widely used because of its efficiency in mapping and recognizing geological contacts and edges. Such traits are of particular importance to archaeological targets.
- 3D Euler Deconvolution (ED): Utilizes both vertical and horizontal derivatives to estimate source boundaries and depth^52,60–62^. It’s very important to determine accurately the parameters that are required when using ED method, such as grid spacing, data spacing, structural index (SI) (geological model) and window size^63^. The structural index (SI) and window size are probably the most essential parameters. The structural index denotes a simplified geological model depending on the local geology (Ibrahim et al., 2022). The greatest depth that can be imaged is determined by the window size, which is approximately half of the maximum depth^63^ .A window size that is too small will prevent the longer wavelength anomalies from being captured making it impossible to estimate their depths^21^.

## Ground penetrating radar (GPR) technique

According to ^**1**^, ground-penetrating radar (GPR) is a vital near-surface geophysical technique widely used in archaeological investigations. GPR is recognized for its exceptionally high resolution in shallow subsurface imaging and its ability to detect anomalies based primarily on contrasts in the dielectric properties of subsurface materials^3^.

In this study, GPR data were acquired at the selected site using the GSSI SIR-4000 system, which is owned by the Geology Department at Sohag University (Fig. 5b). The system was equipped with a 200 MHz central-frequency antenna (Model 5106/A) to ensure high-resolution results. A survey wheel (Model 620, 16-inch diameter) was attached to measure horizontal distances during data acquisition.

**Fig. 7:**
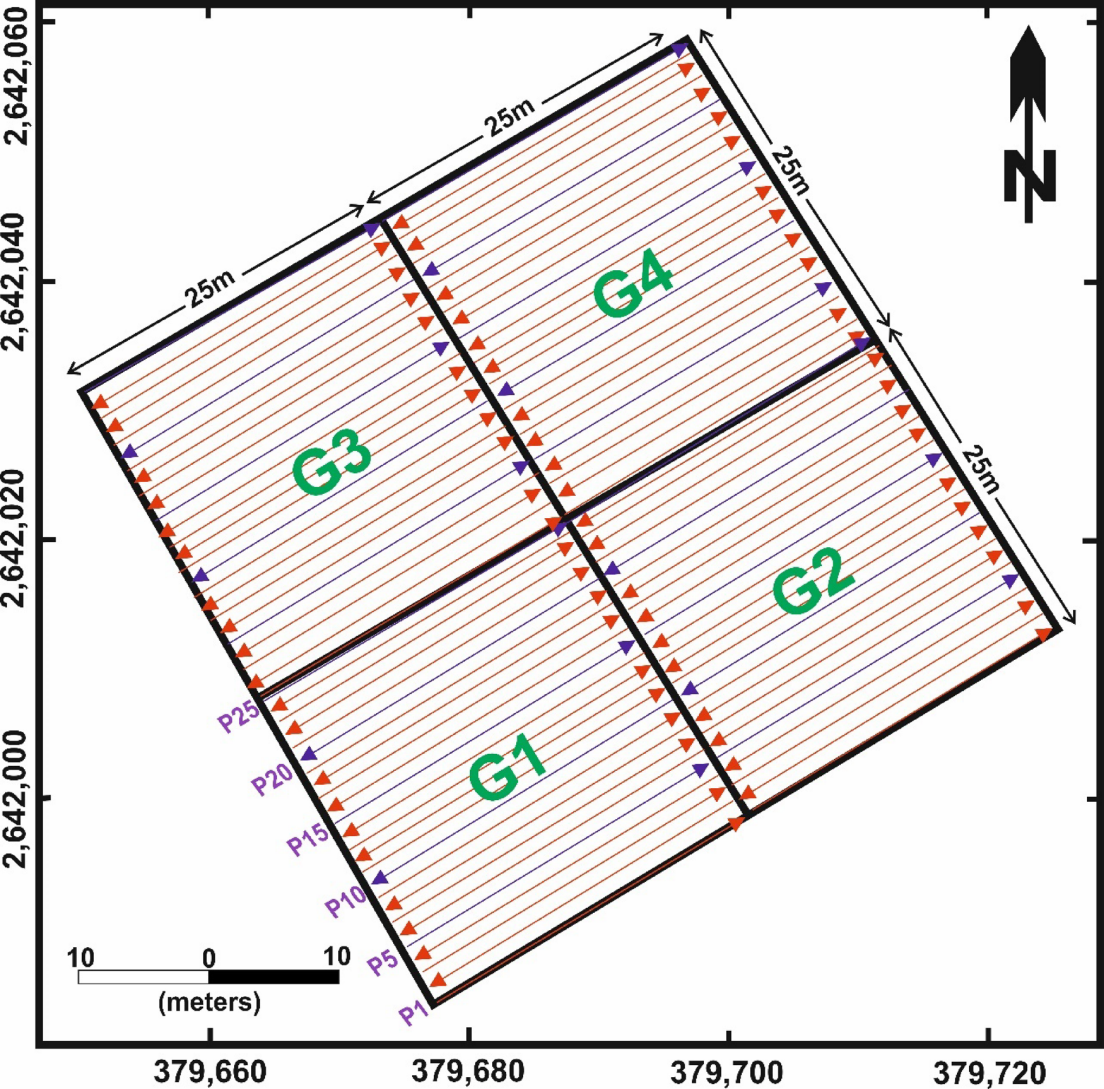
Layout of the Ground Penetrating Radar (GPR) survey at the selected site. The 50 m × 50 m area was subdivided into four blocks (G1–G4), each measuring 25 m × 25 m. Survey profiles (P1–P25) were conducted in a zigzag pattern along evenly spaced lines at 1 m intervals. Coordinate system: WGS 84 / UTM zone 36 N.

The site was subdivided into four equal grids (G1, G2, G3, and G4) (Fig. 7), each measuring 25 × 25 m (625 m²). A total of 104 profiles were collected, 26 profiles per grid, each 25 m long. Data acquisition was conducted in a zigzag pattern along NE–SW oriented lines with 1-meter line spacing.

**Fig. 8:**
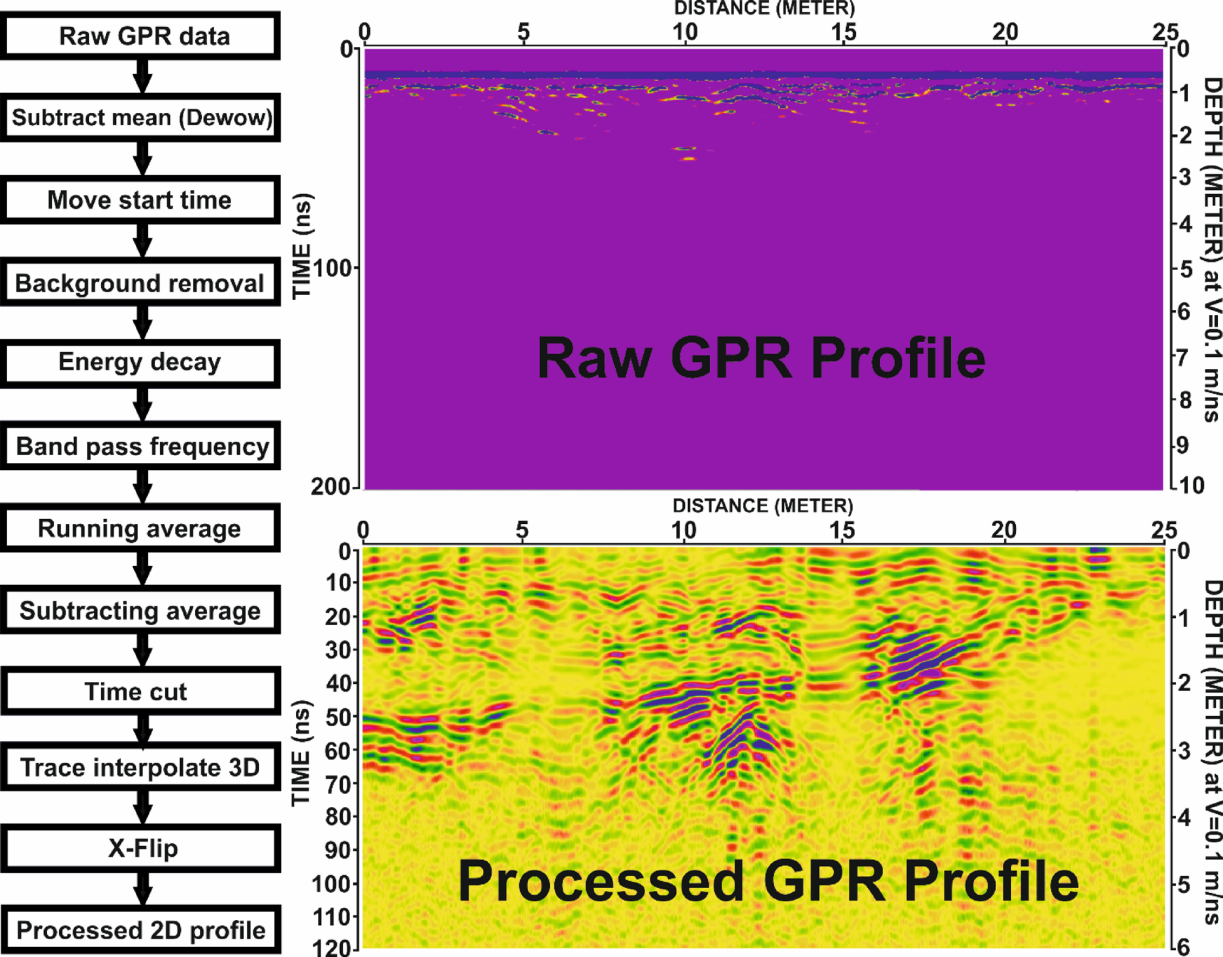
Ground Penetrating Radar (GPR) data processing workflow and example profile. Left: Flowchart illustrating the sequential processing steps applied to the raw GPR data. Right: GPR profile P24 from Grid G3, showing the raw data (top) and the processed 2D profile (bottom), highlighting the enhanced visibility of subsurface features after processing. Depth conversion is based on an assumed velocity of 0.1 m/ns.

Following acquisition, data processing was conducted to remove background noise and suppress unwanted reflections caused by antenna coupling variations, surface clutter, or internal ringing^30^. Processing was carried out using REFLEXW v5.8 software^64^, applying several filtering steps to enhance data clarity (Fig. 8).

To convert the recorded two-way travel time into depth values, the radar wave velocity was calibrated to 0.1 m/ns, which provided appropriate depth resolution for the archaeological context. Several processing steps were applied to the GPR data using REFLEXW v5.8 to enhance data quality and suppress noise:

- Dewow Filter (Subtract Mean): This filter removes low-frequency components by computing a running mean and subtracting it from each trace. It is applied individually to each trace to reduce baseline drift.
- Static Correction (Zero-Time Shift): This correction aligns the vertical position of the first pulse emitted by the antenna, accounting for the separation between the antenna and the ground during movement^3,65^. A shift of 7 ns was applied in this study.

- Background Removal: This step eliminates horizontal banding, direct waves, ringing noise, and random background noise, improving the visibility of subsurface features^66^.
- Gain (Energy Decay) Filter: An automatic amplitude correction was applied using a mean decay curve, compensating for signal attenuation with depth. A scaling factor of 0.2 was used.
- Bandpass Frequency Filter: This filter was applied to remove both low- and high-frequency noise outside the useful frequency range. The frequency settings were 10, 25, 315, and 330 MHz.
- Running Average Filter: Used to suppress trace-dependent noise and emphasize coherent horizontal reflections. The average was applied over 10 traces.
- Subtracting Average Filter: This dynamic background removal filter averages 200 adjacent traces at each time step to eliminate fluctuating background noise across profiles.
- Time Cut: A time window of 120 ns was applied to exclude unnecessary late-time signals, optimize storage, and improve processing efficiency.
- Trace Interpolation: This step adjusted trace density without compromising data integrity, using a trace increment of 0.04 and a profile length of 25 m.
- X-Flip: Since profiles were acquired in a zigzag pattern, this final step reversed the X-direction of alternating lines to ensure spatial continuity. This alignment helps anomalies appear consistent across consecutive radargrams.

## Results and discussion

### Results of magnetic data

**Fig. 9:**
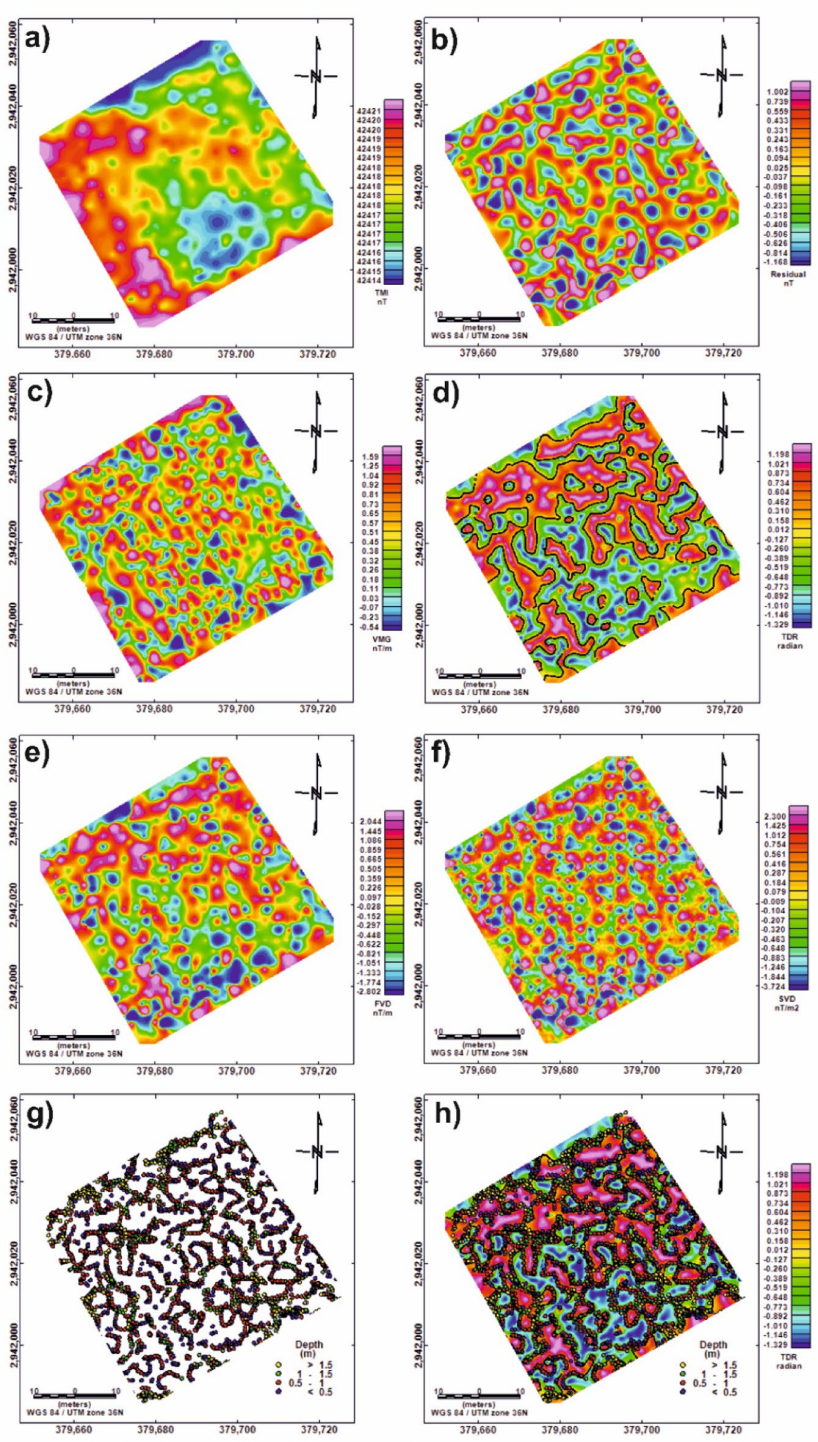
Color maps of magnetic data. (**a**) Total Magnetic Intensity (TMI) map (42,414 to 42,421 nT); (**b**) High-pass filtered (residual) magnetic map (-1.2 to 1.0 nT); (**c**) Vertical Magnetic Gradient (VMG) map (-0.54 to 1.59 nT/m); (**d**) Tilt Derivative (TDR) map with overlaid zero-contour lines (-1.3 and 1.2 radians); (**e**) First Vertical Derivative (FVD) map (-2.8 to 2.0 nT/m); (f) Second Vertical Derivative (SVD) map (-3.7 to 2.4 nT/m²); (g) Euler Deconvolution depth solutions using Structural Index (SI) = 0 (0.2 and 3.0 m); (h) Euler solutions (SI = 0) superimposed on the TDR map for improved interpretation of source locations.

The Total Magnetic Intensity (TMI) map of study site (Fig. 9a) reveals a generally low magnetic gradient, with values ranging from 42,414 to 42,421 nT. The highest magnetic readings are concentrated in the western part of the site, while moderate to low values dominate the rest of the area. To enhance localized anomalies, a Butterworth high-pass filter with a cutoff wavelength of 8 m was applied to the TMI data. The resulting residual magnetic anomaly map (Fig. 9b) displays well-defined circular to semicircular negative anomalies distributed across the site, often bounded by elongated positive zones, that may correspond to mudbrick walls. These anomalies may represent subsurface tombs either dug directly into the sediment or surrounded by mudbrick structures. Residual values range from − 1.2 to 1.0 nT.

The Vertical Magnetic Gradient (VMG) map (Fig. 9c) shows alternating positive and negative anomalies ranging from − 0.54 to 1.59 nT/m. The elongated nature of the positive anomalies suggests the presence of linear features, likely associated with buried walls or architectural remains. Tilt Derivative (TDR) filtering was also applied (Fig. 9d) to highlight the edges of vertical-sided sources using the zero-contour line. TDR values vary between − 1.3 and 1.2 radians.

To further resolve shallow magnetic sources, both the First Vertical Derivative (FVD) and Second Vertical Derivative (SVD) were calculated. The FVD map (Fig. 9e) closely matches the residual anomaly map and highlights near-surface features, with values ranging from − 2.8 to 2.0 nT/m. The SVD map (Fig. 9f), on the other hand, enhances high-frequency components and reveals shallow, small-scale features that may be overlooked by broader filtering methods. SVD values range from − 3.7 to 2.4 nT/m².

Finally, 3D Euler Deconvolution was performed using a structural index (SI) of 0, which is appropriate for identifying vertical contacts. The resulting source depth solutions (Fig. 9g) suggest that the magnetic anomalies originate from depths between 0.2 and 3.0 m, with an average depth of approximately 1 m. These solutions, when superimposed on the TDR map (Fig. 9h), show a strong spatial clustering along the inferred edges of the suspected subsurface features.

### Results of GPR data

The primary objective of GPR surveys in archaeology is to detect the depth, geometry, and position of subsurface remains and associated stratigraphy. GPR data are commonly presented as two-dimensional radargrams or reflection profiles. Reflections from buried features typically appear as hyperbolic or linear patterns, depending on the orientation of the antenna relative to the geometry of the target^66^.

**Fig. 10:**
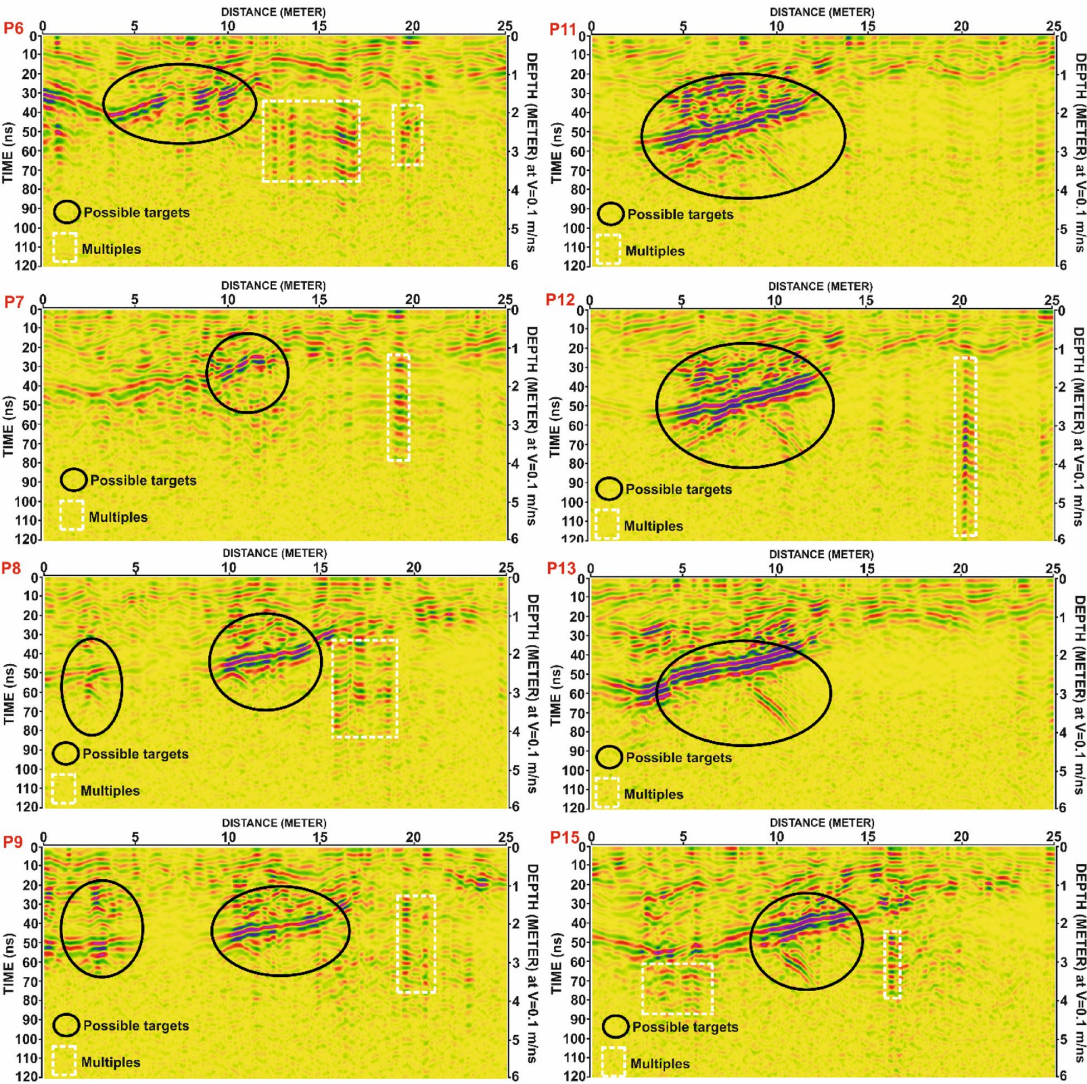
Processed Ground Penetrating Radar (GPR) profiles from Grid G1 for lines P6, P7, P8, P9, P11, P12, P13, and P15. Black ellipses indicate zones of possible subsurface anomalies (potential archaeological targets), while white dashed rectangles highlight areas interpreted as multiples (signal reflections from strong interfaces). Time is shown in nanoseconds (ns), with corresponding depth estimated using a velocity of 0.1 m/ns.

The processed GPR profiles from Grid G1 are shown in Fig. 10, with representative examples selected from profiles P6–P9 and P11–P13, in addition to P15. A prominent archaeological anomaly was consistently detected across several of these profiles:

- In Profile P6, the anomaly appears between 4 and 10 m along the surface, with a maximum horizontal extent of 6 m. It is located at depths ranging from 1.2 to 2.2 m, with an amplitude of 1 m and a two-way travel time (TWT) between 24 and 44 ns.
- In Profile P7, the same anomaly is observed between 9 and 12 m, extending 3 m horizontally, with a depth of 1.0–1.8 m, amplitude of 0.8 m, and a TWT of 20–36 ns.
- In Profile P8, it is detected between 10 and 14 m, with a horizontal width of 4 m, depth range of 1.7–2.8 m, amplitude of 1.1 m, and TWT of 34–56 ns. Additionally, a second anomaly is observed between 1 and 4 m, with a horizontal extent of 3 m, depth of 2.2–3.2 m, amplitude of 1 m, and TWT of 44–64 ns.
- In Profile P9, two distinct anomalies were recorded. The first spans from 2 to 3.5 m, with a horizontal length of 1.5 m, depth of 1.2–3.0 m, amplitude of 1.8 m, and TWT of 24–60 ns. The second anomaly appears between 10 and 15 m, with a 5-meter width, depth range of 1.8–2.8 m, amplitude of 1 m, and TWT of 36–56 ns.

Additional prominent reflections were found in other profiles:

- Profile P11 shows a broad anomaly from 4 to 12 m along the surface, with a horizontal span of 8 m, depth of 1.2–3.0 m, amplitude of 1.8 m, and TWT between 24 and 60 ns.
- In Profile P12, the anomaly extends from 5 to 12 m, covering 7 m horizontally, at depths of 1.5–3.7 m, amplitude of 2.2 m, and TWT from 30 to 74 ns.
- Profile P13 reveals an anomaly from 2.5 to 10 m, with a horizontal extent of 7.5 m, depth range of 1.8–4.0 m, amplitude of 2.2 m, and TWT of 36–80 ns.
- In Profile P15, the anomaly is recorded between 9 and 14 m, with a horizontal dimension of 5 m, depth of 1.7–3.5 m, amplitude of 1.8 m, and TWT ranging from 34 to 70 ns.

**Fig. 11:**
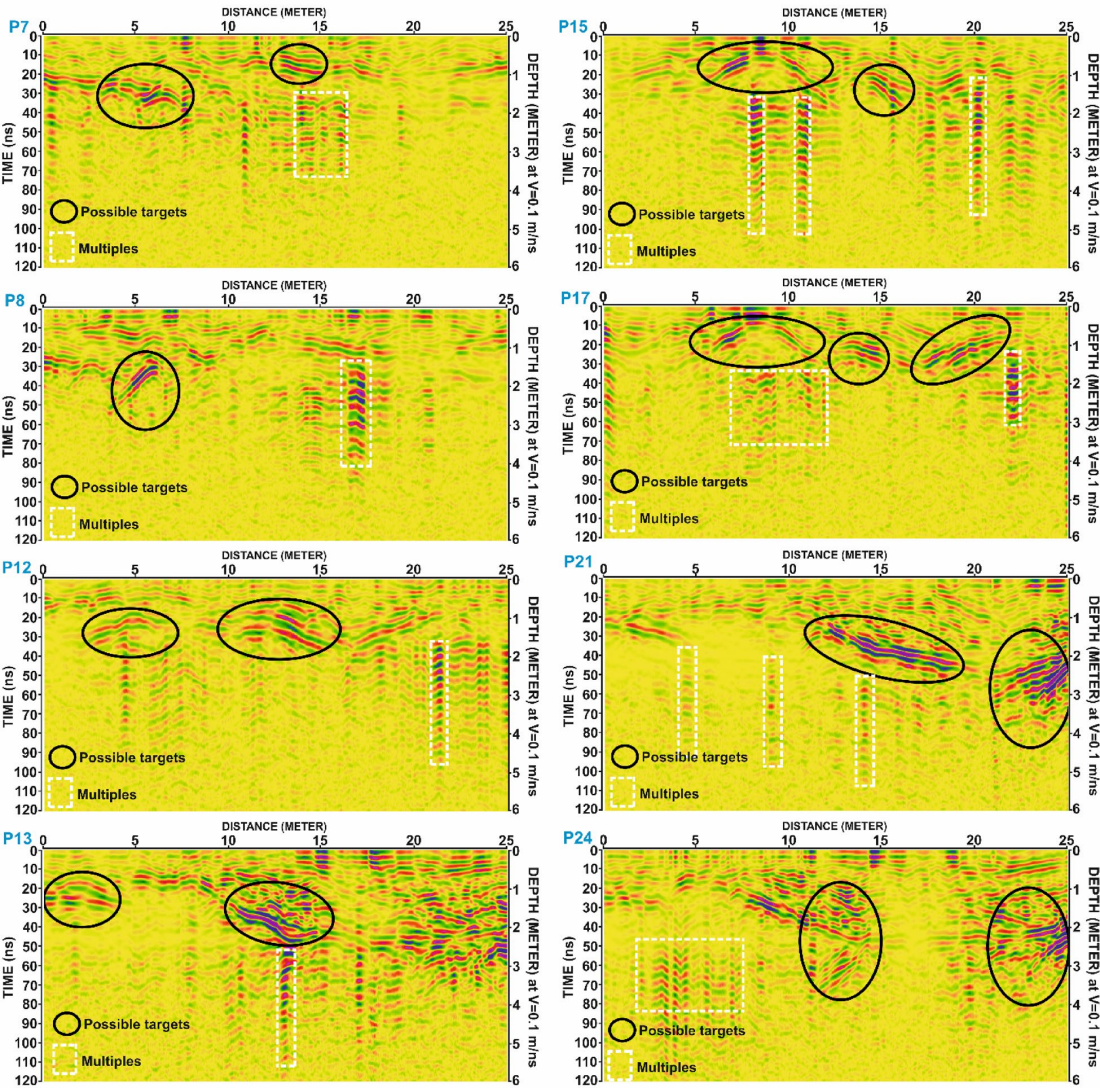
Processed Ground Penetrating Radar (GPR) profiles from Grid G2 for lines P7, P8, P12, P13, P15, P17, P21, and P24. Black ellipses indicate potential subsurface anomalies interpreted as possible archaeological targets. White dashed rectangles mark locations of multiple reflections. Depth conversion is based on an assumed velocity of 0.1 m/ns.

**Fig. 12:**
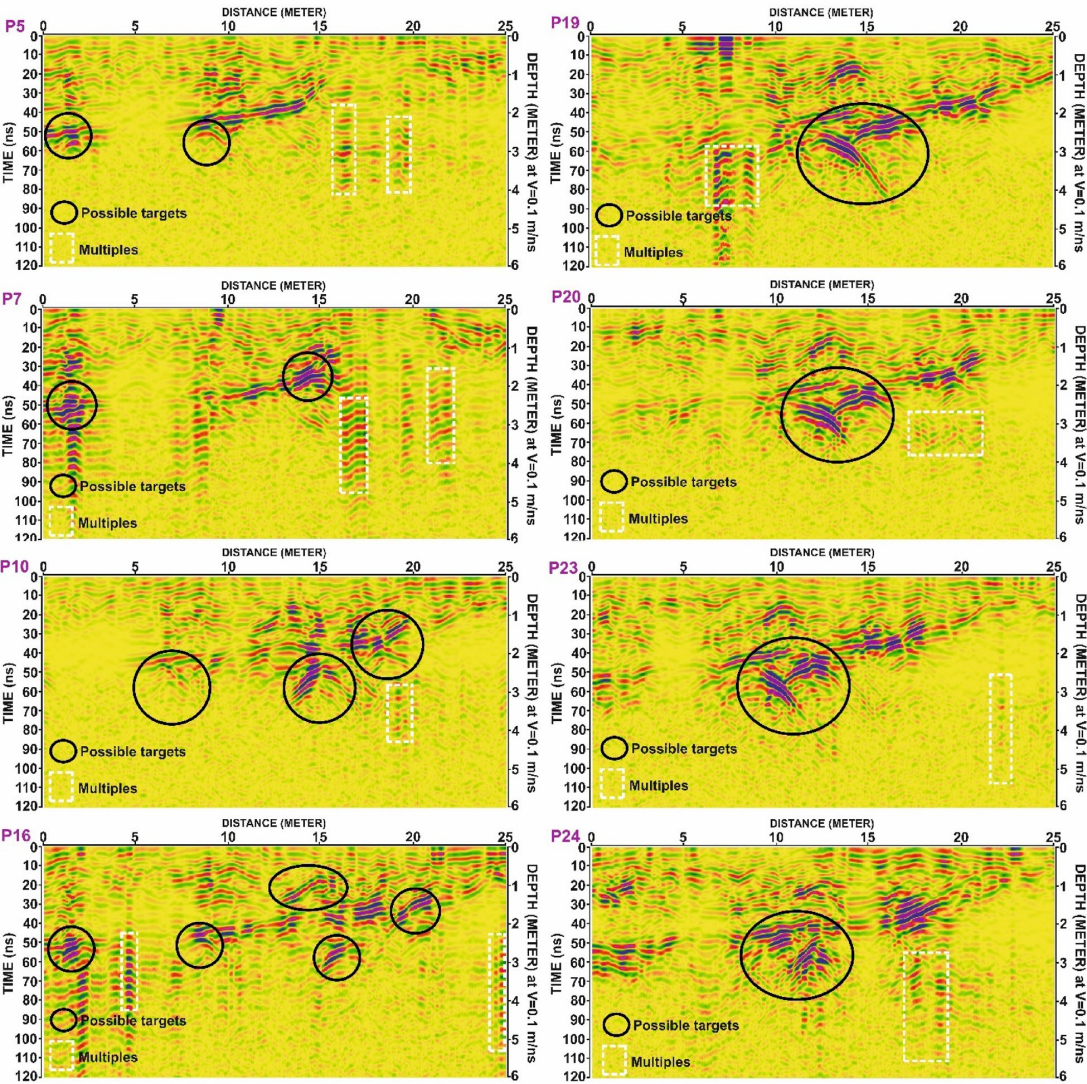
Processed Ground Penetrating Radar (GPR) profiles from Grid G3 for lines P5, P7, P10, P16, P19, P20, P23, and P24. Black ellipses denote zones of interest interpreted as possible subsurface targets. White dashed rectangles highlight areas identified as multiples. Depth scale is based on an assumed electromagnetic wave velocity of 0.1 m/ns.

**Fig. 13:**
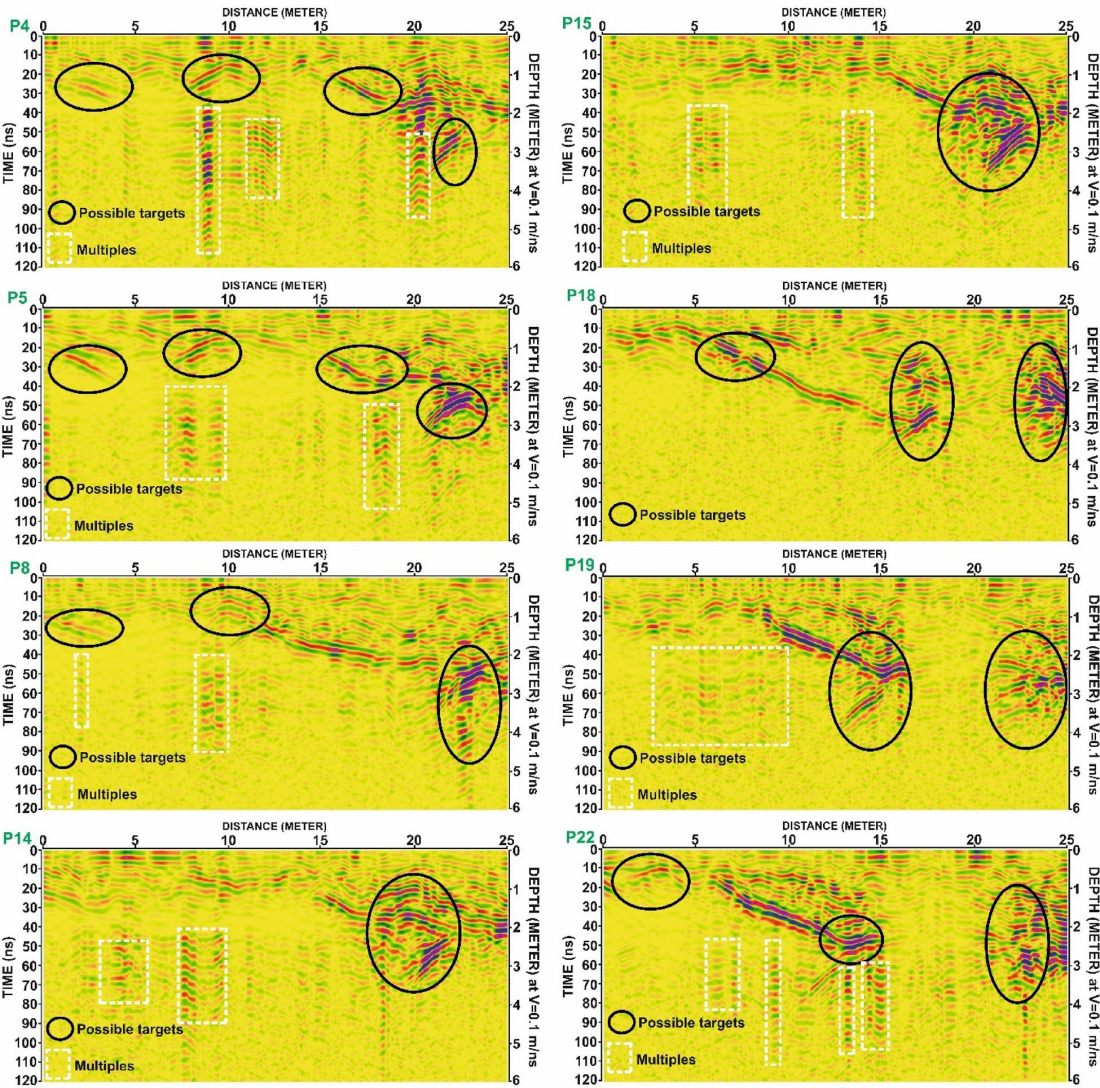
Processed Ground Penetrating Radar (GPR) profiles from Grid G4 for lines P4, P5, P8, P14, P15, P18, P19, and P22. Black ellipses mark potential subsurface anomalies interpreted as possible archaeological targets. White dashed rectangles indicate zones of multiple reflections. Depth scale is based on an assumed wave velocity of 0.1 m/ns.

To maintain conciseness in the manuscript, only the results from Grid G1 are described in detail. The findings from Grids G2, G3, and G4 are summarized in Tables 2, 3, 4 and 5. Selected examples of GPR anomalies in:

- Grid G2 are illustrated in Fig. 11, including profiles P7, P8, P12, P13, P15, P17, P21, and P24.
- Grid G3 are presented in Fig. 12, highlighting reflections in profiles P5, P7, P10, P19, P20, P23, and P24.
- Grid G4 are shown in Fig. 13, including profiles P4, P5, P8, P14, P15, P18, P19, and P22.

**Table 2:** The parameters of the potential anomalies that are detected in G1 grid.

Profile No.	Archaeological Anomaly	Surface Distance (m)	Maximum Horizontal Dimension (m)	Depth to the Top and Bottom (m)	Amplitude (m)	Two-Way Time (ns)
P6	1	04 to 10	6.0	1.2 to 2.2	1.0	24 to 44
P7	1	09 to 12	3.0	1.0 to 1.8	0.8	20 to 36
P8	1	01 to 04	3.0	2.2 to 3.2	1.0	44 to 64
	2	10 to 14	4.0	1.7 to 2.8	1.1	34 to 56
P9	1	02 to 3.5	1.5	1.2 to 3.0	1.8	24 to 60
	2	10 to 15	5.0	1.8 to 2.8	1.0	36 to 56
P11	1	04 to 12	8.0	1.2 to 3.0	1.8	24 to 60
P12	1	05 to 12	7.0	1.5 to 3.7	2.2	30 to 74
P13	1	2.5 to 10	7.5	1.8 to 4.0	2.2	36 to 80
P15	1	09 to 14	5.0	1.7 to 3.5	1.8	34 to 70

**Table 3:** The parameters of the potential anomalies that are detected in G2 grid.

Profile No.	Archaeological Anomaly	Surface Distance (m)	Maximum Horizontal Dimension (m)	Depth to the Top and Bottom (m)	Amplitude (m)	Two-Way Time (ns)
P7	1	04.0 to 07.5	3.5	1.0 to 2.0	1.0	20 to 40
	2	12.5 to 15.0	2.5	0.4 to 1.2	0.8	08 to 24
P8	1	04.0 to 06.5	2.5	1.3 to 2.5	1.2	26 to 50
P12	1	02.5 to 06.5	4.0	0.8 to 1.8	1.0	16 to 36
	2	10.0 to 15.0	5.0	0.7 to 1.8	1.1	14 to 36
P13	1	01.0 to 03.0	2.0	0.6 to 1.7	1.1	12 to 34
	2	10.0 to 15.0	5.0	1.0 to 2.2	1.2	20 to 44
P15	1	06.0 to 12.0	6.0	0.3 to 1.2	0.9	06 to 24
	2	14.0 to 16.0	2.0	1.0 to 2.0	1.0	20 to 40
P17	1	06.0 to 12.0	6.0	0.3 to 1.2	0.9	06 to 24
	2	12.5 to 15.0	2.5	0.9 to 1.7	0.8	18 to 34
	3	17.0 to 22.0	5.0	0.8 to 1.7	0.9	16 to 34
P21	1	12.0 to 18.0	6.0	1.2 to 2.3	1.1	24 to 46
	2	22.0 to 25.0	3.0	2.0 to 3.0	1.0	40 to 60
P24	1	12.0 to 15.0	3.0	1.0 to 3.0	2.0	20 to 60
	2	22.0 to 25.0	3.0	1.8 to 3.0	1.2	36 to 60
	5	18.0 to 21.0	3.0	1.2 to 2.0	0.8	24 to 40

**Table 4:** The parameters of the potential anomalies that are detected in G3 grid.

Profile No.	Archaeological Anomaly	Surface Distance (m)	Maximum Horizontal Dimension (m)	Depth to the Top and Bottom (m)	Amplitude (m)	Two-Way Time (ns)
P5	1	01.0 to 02.5	1.5	2.2 to 3.0	0.8	44 to 60
	2	07.5 to 10.0	2.5	2.2 to 3.0	0.8	44 to 60
P7	1	01.0 to 02.5	1.5	2.2 to 3.0	0.8	44 to 60
	2	13.0 to 15.0	2.0	1.5 to 2.2	0.7	30 to 44
P10	1	06.0 to 08.0	2.0	2.1 to 3.0	0.9	42 to 60
	2	13.5 to 17.0	3.5	2.2 to 3.5	1.3	44 to 70
	3	17.5 to 20.0	2.5	1.0 to 2.2	1.2	20 to 44
P16	1	01.0 to 02.5	1.5	2.2 to 3.0	0.8	44 to 60
	2	07.5 to 09.0	1.5	2.0 to 3.0	1.0	40 to 60
	3	12.5 to 16.0	3.5	0.7 to 1.5	0.8	14 to 30
	4	15.0 to 16.5	1.5	2.5 to 3.0	0.5	50 to 60
	5	18.0 to 21.0	3.0	1.2 to 2.0	0.8	24 to 40
P19	1	12.0 to 16.0	4.0	2.0 to 4.0	2.0	40 to 80
P20	1	12.0 to 16.0	4.0	2.0 to 3.8	1.8	40 to 76
P23	1	08.0 to 13.0	5.0	2.0 to 3.8	1.8	40 to 76
P25	1	08.0 to 12.5	4.5	1.9 to 3.5	1.6	28 to 70

**Table 5:** The parameters of the potential anomalies that are detected in G4 grid.

Profile No.	Archaeological Anomaly	Surface Distance (m)	Maximum Horizontal Dimension (m)	Depth to Its Top and Bottom (m)	Amplitude (m)	Two-Way Time (ns)
P4	1	01.0 to 04.0	3.0	0.8 to 1.8	1.0	16 to 36
	2	08.0 to 11.0	3.0	0.6 to 1.5	0.9	12 to 30
	3	16.0 to 18.0	4.0	1.0 to 1.8	0.8	20 to 36
	4	21.0 to 23.0	2.0	2.4 to 3.5	1.1	48 to 70
P5	1	01.0 to 04.0	3.0	0.8 to 1.8	1.0	16 to 36
	2	08.0 to 10.0	2.0	0.6 to 1.5	0.9	12 to 30
	3	16.0 to 18.0	2.0	1.0 to 1.8	0.8	20 to 36
	4	21.0 to 23.0	2.0	2.4 to 3.5	1.1	48 to 70
P8	1	01.0 to 04.0	3.0	1.0 to 1.8	0.8	20 to 36
	2	09.0 to 12.0	3.0	0.4 to 1.2	0.8	08 to 24
	3	21.0 to 24.0	3.0	2.0 to 3.5	1.5	40 to 70
P14	1	17.5 to 21.0	3.5	1.0 to 3.2	2.2	20 to 64
P15	1	18.0 to 23.0	5.0	1.0 to 3.5	2.5	20 to 70
P18	1	06.0 to 08.0	2.0	0.5 to 1.7	1.2	10 to 34
	2	16.0 to 18.0	2.0	2.2 to 3.2	1.0	44 to 64
	3	22.5 to 25.0	2.5	1.7 to 3.0	1.3	34 to 60
P19	1	14.0 to 16.0	2.0	2.0 to 3.5	1.5	40 to 70
	2	22.5 to 25.0	2.5	2.2 to 3.5	1.3	44 to 70
P22	1	01.0 to 05.0	4.0	0.3 to 0.9	0.6	06 to 18
	2	12.5 to 15.0	2.5	2.1 to 2.9	0.8	42 to 58
	3	22.0 to 24.0	2.0	1.5 to 3.0	1.5	30 to 60

## Integration of magnetic and GPR data

The magnetic results, particularly the First Vertical Derivative (FVD) map, were integrated with ground penetrating radar (GPR) data to enhance the interpretation of subsurface archaeological features. This integration aimed to validate and cross-reference anomalies detected by both techniques and to improve the reliability of interpretations.

**Fig. 14:**
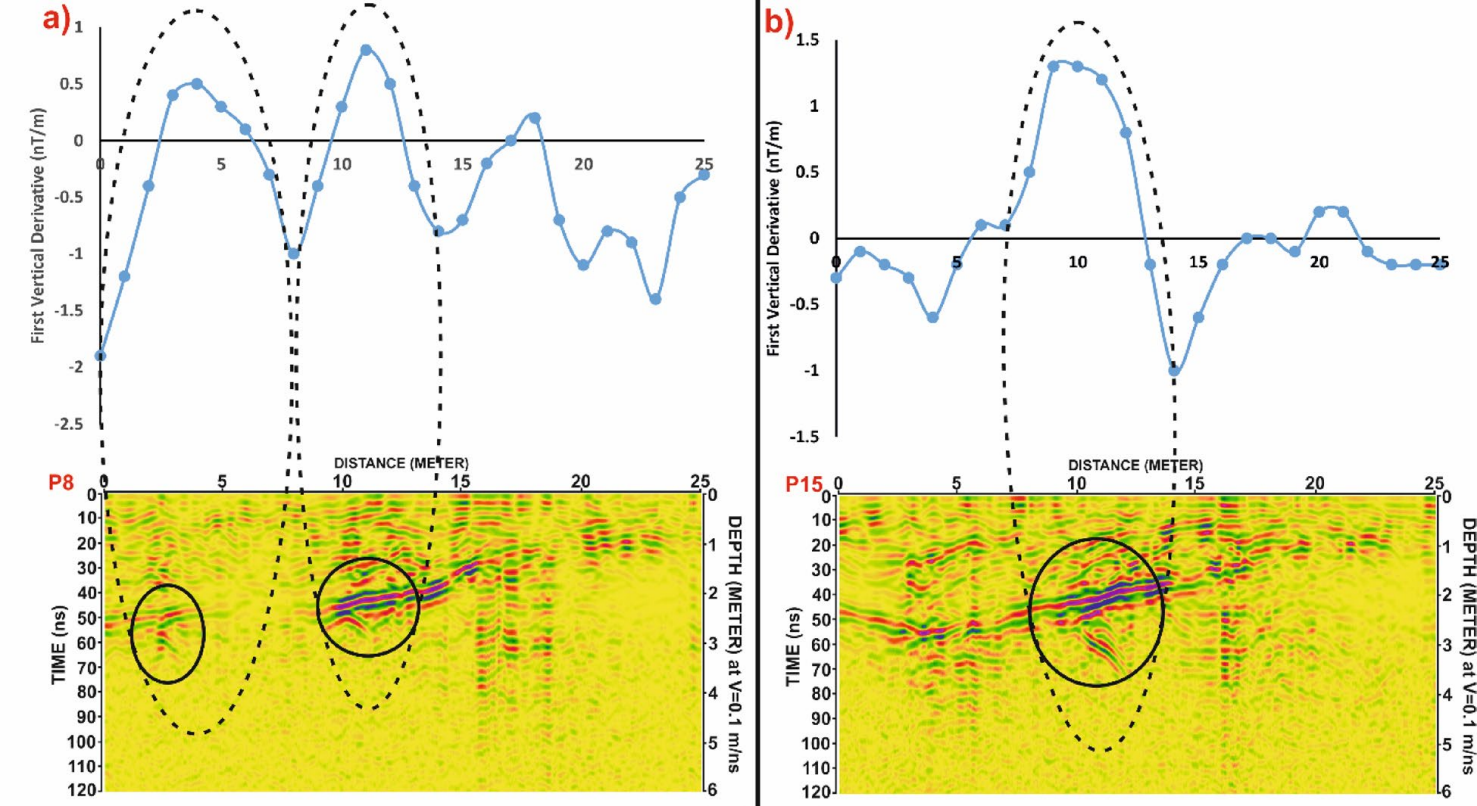
Correlation between magnetic and GPR datasets for selected profiles from Grid G1. (**a**) Profile P8 and (**b**) Profile P15, showing the first vertical derivative (FVD) magnetic data (top) and corresponding processed GPR sections (bottom). Black dashed ellipses highlight zones of spatial correspondence between magnetic anomalies and potential subsurface targets identified in the GPR data. Depth scale is based on a velocity of 0.1 m/ns.

**Fig. 15:**
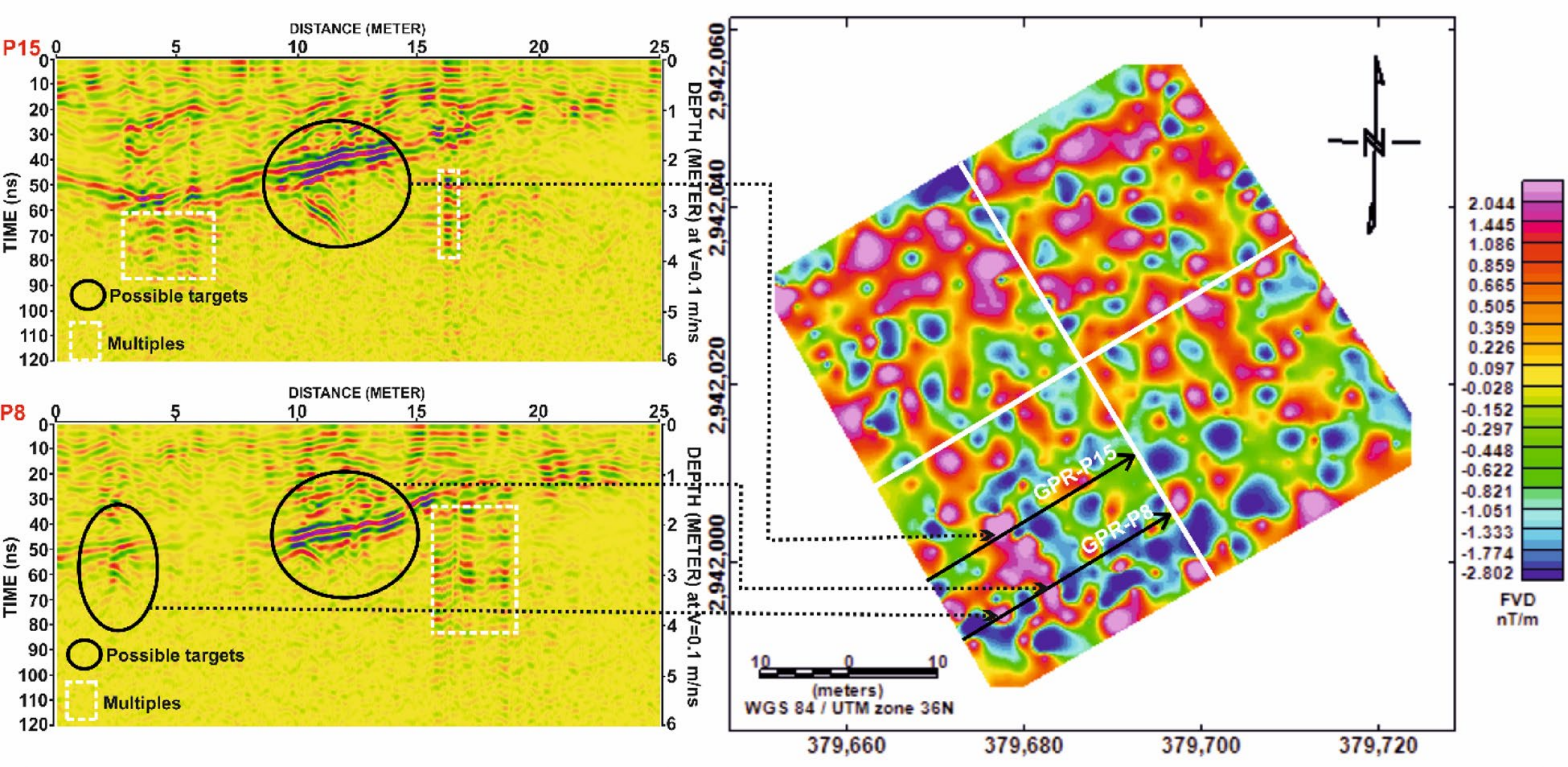
Correlation between Ground Penetrating Radar (GPR) profiles (P8 and P15) from Grid G1 and the magnetic anomalies identified in the First Vertical Derivative (FVD) map of study site. The spatial alignment of GPR-detected subsurface targets with corresponding magnetic anomalies demonstrates strong agreement in delineating potential archaeological features. Dashed lines link interpreted anomalies across both datasets, reinforcing the reliability of integrated geophysical interpretation.

In Grid G1, Fig. 14 presents a profile-based comparison of FVD and GPR data for profiles P8 and P15. A strong spatial correspondence is observed between the positive magnetic anomalies and the hyperbolic reflections in the GPR profiles, suggesting the presence of linear structures, most likely mudbrick walls, potentially forming parts of tomb complexes. Figure 15 further supports this interpretation by correlating these profiles spatially within the FVD map, reinforcing the consistency of the identified anomalies across both methods.

**Fig. 16:**
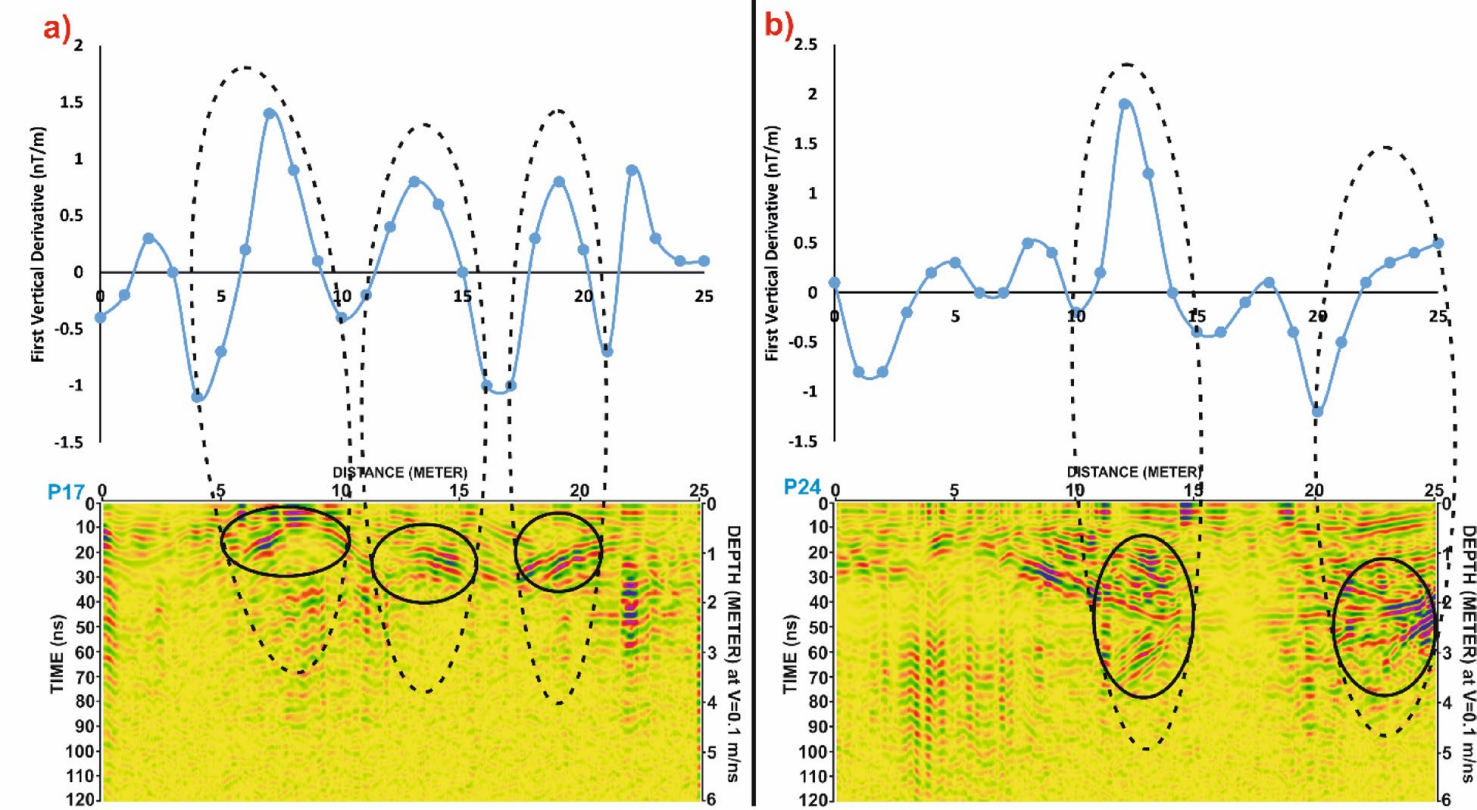
Comparative analysis of magnetic and GPR data for Grid G2. (**a**) Profile P17 and (**b**) Profile P24, showing the First Vertical Derivative (FVD) magnetic data (top) and corresponding processed GPR sections (bottom). Black dashed ellipses highlight correlated anomalies in both datasets, supporting the interpretation of potential subsurface archaeological features. Depth estimates in GPR profiles are based on an assumed velocity of 0.1 m/ns.

**Fig. 17:**
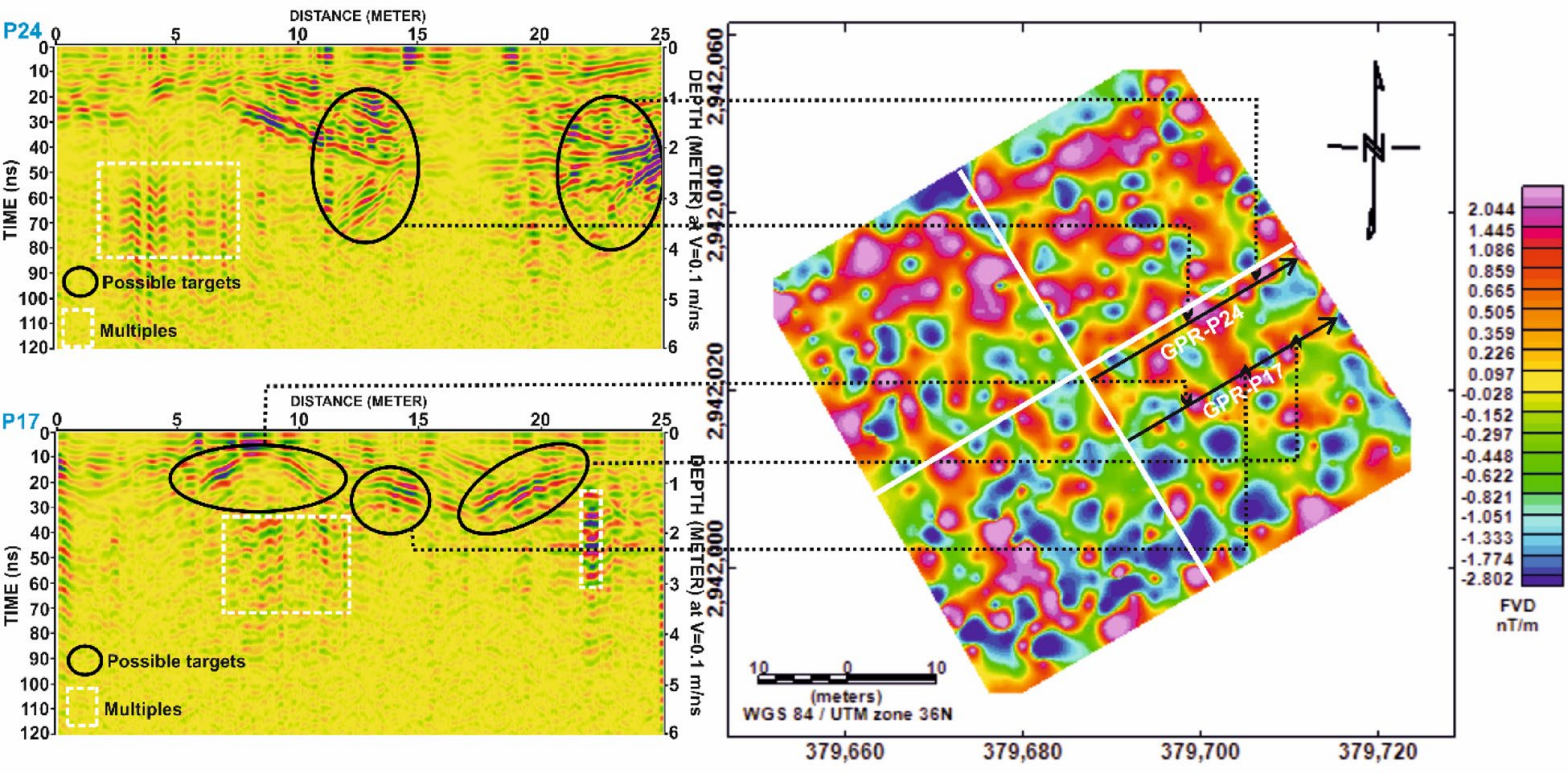
Correlation of Ground Penetrating Radar (GPR) profiles P17 and P24 from Grid G2 with magnetic anomalies represented by the First Vertical Derivative (FVD) map of study site. Dashed lines connect zones of interest between datasets, showing strong spatial agreement in the delineation of possible archaeological features. Black ellipses indicate potential targets detected in GPR sections, corresponding with prominent magnetic anomalies in the FVD map.

Similarly, in Grid G2, Fig. 16 compares profiles P17 and P24, showing clear alignment between magnetic highs and GPR reflections. The matched anomalies further support the interpretation of buried architectural elements, possibly including wall foundations or other constructed features. Figure 17 extends this correlation by illustrating the spatial footprint of the anomalies, confirming the presence of subsurface structures in both datasets.

**Fig. 18:**
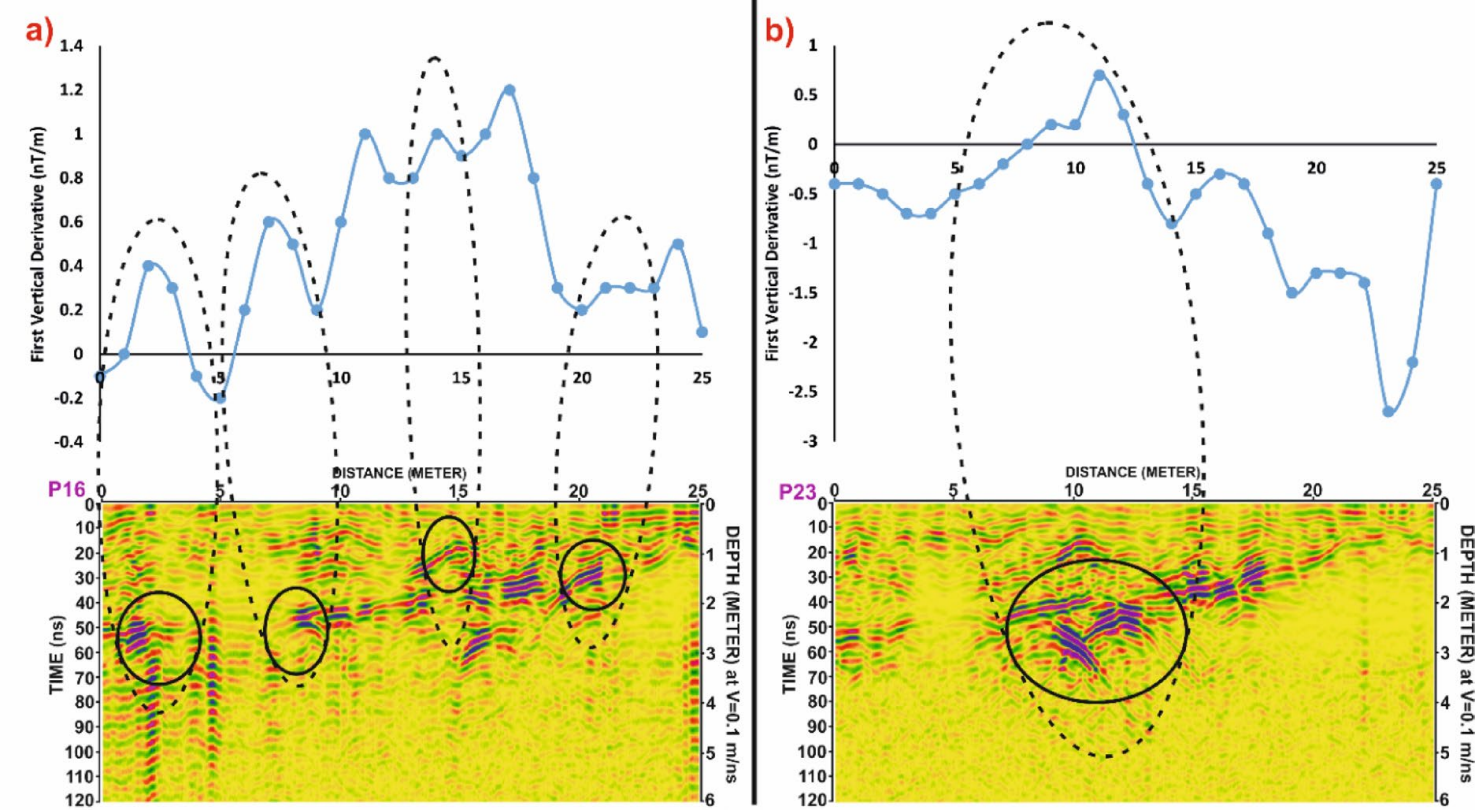
Comparative analysis of magnetic and GPR data for Grid G3. (**a**) Profile P16 and (**b**) Profile P23, showing First Vertical Derivative (FVD) magnetic anomalies (top) and corresponding processed Ground Penetrating Radar (GPR) profiles (bottom). Dashed black ellipses outline correlated zones where magnetic anomalies align with GPR-detected reflections, indicating potential subsurface archaeological features. Depth estimates are based on an assumed velocity of 0.1 m/ns.

**Fig. 19:**
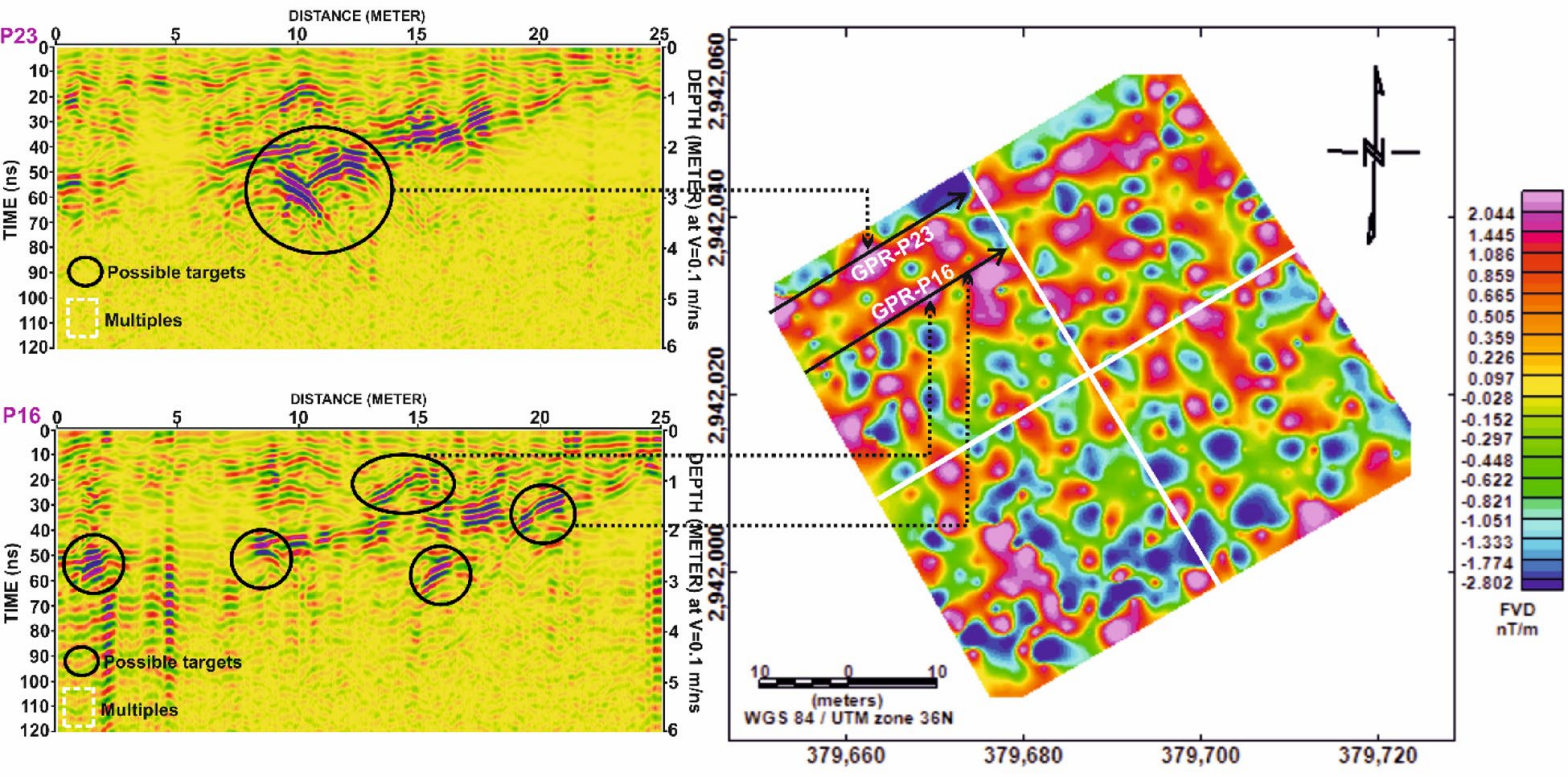
Correlation of Ground Penetrating Radar (GPR) profiles P16 and P23 from Grid G3 with magnetic anomalies depicted in the First Vertical Derivative (FVD) map of study site. Black ellipses in the GPR sections highlight potential subsurface targets, while dashed lines link them to corresponding magnetic anomalies, demonstrating strong agreement between both datasets in identifying possible archaeological features. Coordinate system: WGS 84 / UTM zone 36 N.

In Grid G3, Fig. 18 compares profiles P16 and P23, where prominent magnetic highs correlate well with distinct GPR hyperbolae. This alignment indicates potential subsurface structures, likely related to burial architecture. However, Fig. 19 reveals that certain reflections in the GPR profile of P16 lack corresponding magnetic anomalies. These features may be composed of materials with low magnetic susceptibility, such as limestone sarcophagi or organic remains, which may not produce significant magnetic responses.

**Fig. 20:**
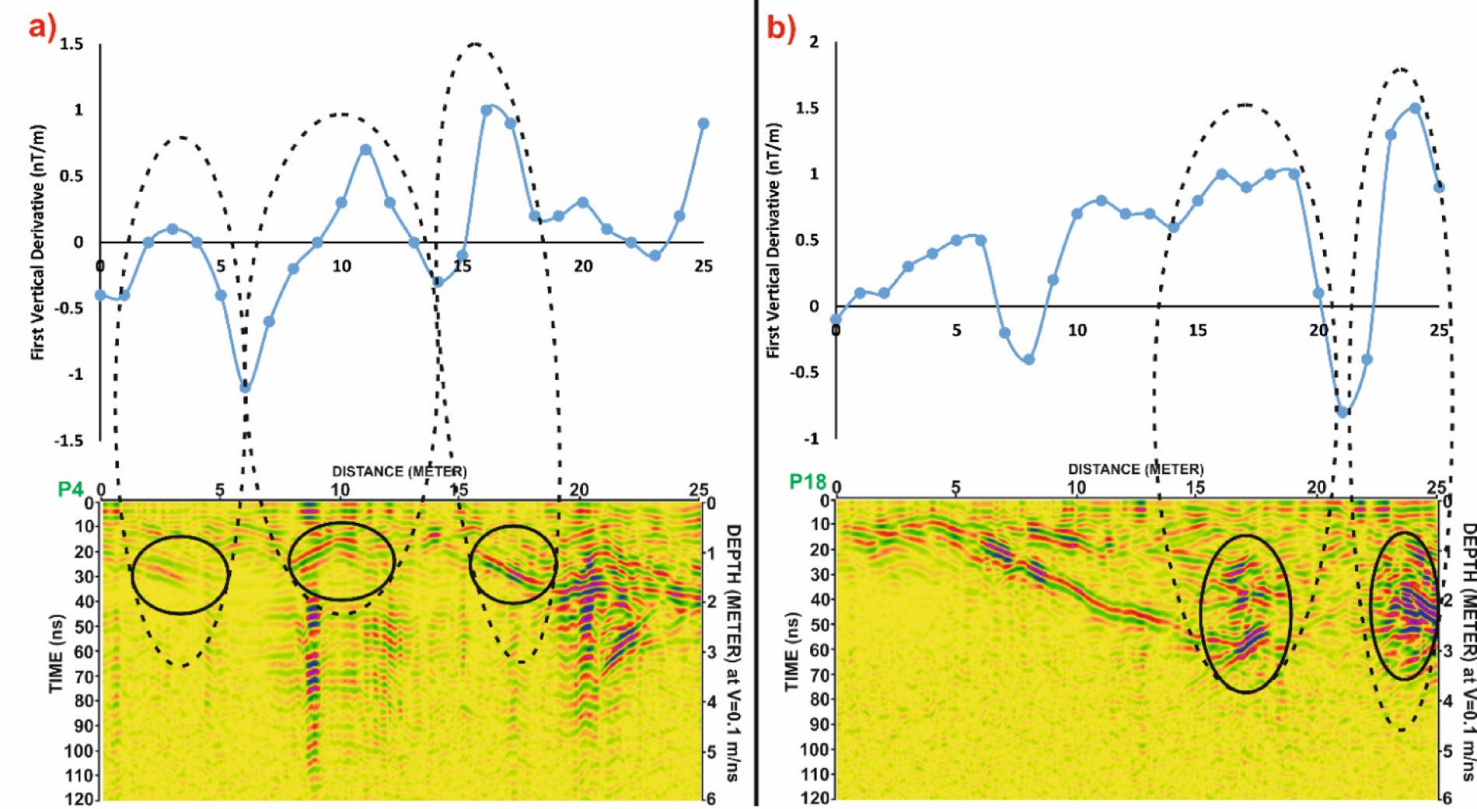
Comparison of magnetic and GPR data for Grid G4. (**a**) Profile P4 and (**b**) Profile P18, displaying First Vertical Derivative (FVD) magnetic anomalies (top) alongside corresponding processed Ground Penetrating Radar (GPR) sections (bottom). Dashed ellipses highlight areas of spatial correlation between the magnetic signals and radar reflections, indicating potential subsurface archaeological features. Depth conversion in GPR sections assumes a velocity of 0.1 m/ns.

**Fig. 21:**
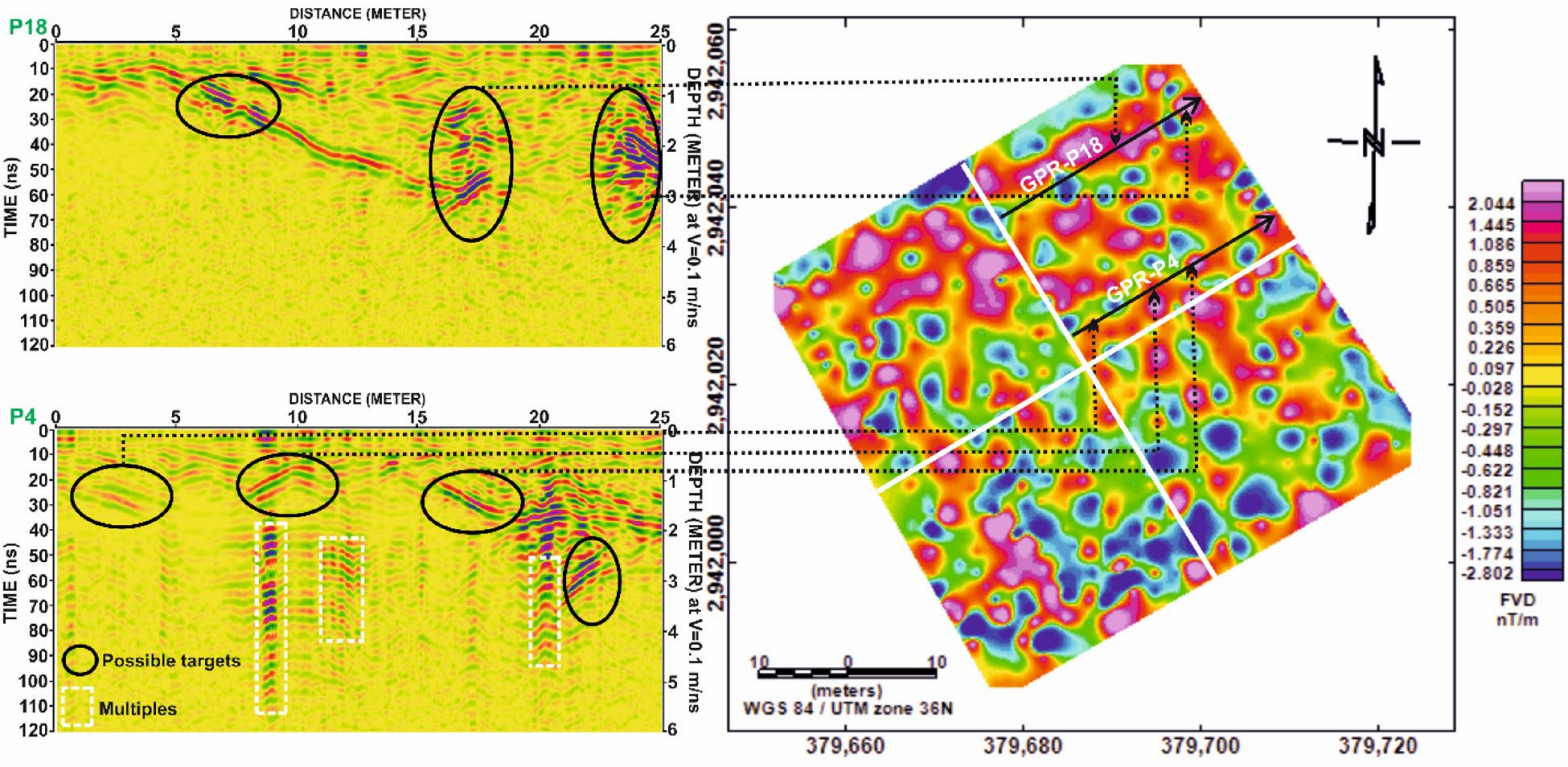
Correlation of Ground Penetrating Radar (GPR) profiles P4 and P18 from Grid G4 with magnetic anomalies shown in the First Vertical Derivative (FVD) map of study site. Dashed black lines connect potential subsurface features identified in the GPR data (highlighted by black ellipses) to corresponding magnetic anomalies, demonstrating strong agreement in the identification of possible archaeological structures. White dashed rectangles in the GPR profiles indicate multiples. Coordinate system: WGS 84 / UTM zone 36 N.

In Grid G4, Fig. 20 continues the comparison for profiles P4 and P18, again revealing a strong correspondence between magnetic anomalies and GPR reflections. Figure 21 reinforces this interpretation by showing that the spatial distribution of anomalies aligns across both datasets, confirming their archaeological relevance.

Overall, the integrated interpretation across Figs. 14, 15, 16, 17, 18, 19, 20 and 21 demonstrates the strength of a multi-method geophysical approach. Magnetic methods provided valuable insight into lateral extents and contrasts in magnetic susceptibility, while GPR added resolution in terms of depth, geometry, and internal structure. Together, these techniques proved complementary in detecting and characterizing buried archaeological features, increasing both confidence and accuracy in subsurface interpretation.

A comparative analysis was conducted across Figs. 14, 16 and 18, and 20, (Table 6) which displays the correlation between First Vertical Derivative (FVD) magnetic data and Ground Penetrating Radar (GPR) results along profiles from Grids G1 to G4. The objective was to assess the degree of alignment between magnetic anomalies and radar-detected subsurface reflections interpreted as potential archaeological features.

**Table 6:** Comparative summary of correlation between FVD magnetic anomalies and GPR profiles across grids G1–G4. This table highlights the number of identified targets, degree of agreement between magnetic and radar data, estimated depths, and interpretation confidence for each pair of profiles from the respective survey grids.

Parameter	Figure 14 (P8 and P15, Grid G1)	Figure 16 (P17 and P24, Grid G2)	Figure 18 (P16 and P23, Grid G3)	Figure 20 (P4 and P18, Grid G4)
Number of interpreted targets	2 in P8, 1 in P15	3 in P17, 2 in P24	4 in P16, 1 in P23	3 in P4, 2 in P18
FVD–GPR agreement quality	Strong and consistent in both profiles	Good alignment, especially in P17	Very strong in P16, moderate in P23	Clear and reliable, especially in P4
Estimated target depth	Approximately 0.5–2.5 m	Approximately 0.5–2.0 m	Approximately 0.5–3.0 m	Approximately 0.5–2.5 m
Magnetic signature quality	Well-defined and matches GPR targets	Repetitive and aligned in P17; less clear in P24	Strong and coherent in P16	Spatially consistent with radar anomalies
GPR reflection complexity	Moderate; features are interpretable	High complexity in P17; moderate in P24	Complex and possibly overlapping targets	Moderate to high; well-separated anomalies
Interpretive confidence	High	Moderate to high	High, with multi-anomaly support	High

The combined analysis of FVD magnetic anomalies and GPR data confirms that integrated geophysical interpretation significantly enhances confidence in identifying shallow archaeological targets. The highest correlation was observed in Grids G1 and G3, supporting the value of multi-method approaches in non-invasive subsurface investigations.

To further assess the spatial consistency between the magnetic and radar datasets, a comparative analysis was conducted between the GPR profiles and their corresponding First Vertical Derivative (FVD) magnetic anomaly maps. Figures 15, 17 and 19, and 21 illustrate this correlation across Grids G1 to G4. The summary provided in Table 7 highlights the degree of alignment, interpretive clarity, and reliability of the detected subsurface features using the integrated geophysical approach.

**Table 7:** Comparative summary of Spatial correlation between GPR profiles and magnetic anomalies (FVD maps) across grids G1–G4. This table presents the degree of agreement between radar-detected subsurface features and magnetic anomalies, the clarity of Spatial alignment, and interpretation reliability for profiles from grids G1 to G4 (Figs. 15, 17 and 19, and 21).

Parameter	Figure 15 (P8 and P15, Grid G1)	Figure 17 (P17 and P24, Grid G2)	Figure 19 (P16 and P23, Grid G3)	Figure 21 (P4 and P18, Grid G4)
Number of GPR profiles	2 (P8 and P15)	2 (P17 and P24)	2 (P16 and P23)	2 (P4 and P18)
Grid location	G1	G2	G3	G4
GPR–FVD spatial alignment	Very clear in both profiles	Strong in P17, moderate in P24	Excellent in P16, less in P23	Strong in both profiles
Number of matched anomalies	2–3 per profile	2–3 in P17, 1–2 in P24	Up to 4 in P16, 1–2 in P23	2–3 in both profiles
Visual clarity of correlation	High	Moderate	Very high	High
Use of reference lines/arrows	Present and effective	Present but limited in P24	Present and well-positioned	Clear and consistent
Interpretive confidence	High	Moderate to high	Very high in P16; moderate in P23	High

The spatial integration of GPR and magnetic data across the four grids demonstrates the effectiveness of a multi-method geophysical approach for archaeological feature detection. The clearest and most reliable correlations are observed in Grids G1 and G3 (Figs. 17 and 21), underscoring their potential as priority zones for future excavation.

## Conclusion

The integrated magnetic and GPR survey conducted at Al-Dyabat hill has successfully demonstrated the potential of geophysical techniques in non-invasively identifying and delineating subsurface archaeological features. The magnetic survey clearly revealed multiple previously undocumented features, including tomb-like structures and possible limestone coffins. GPR data added crucial depth and material differentiation, particularly identifying non-magnetic features invisible in the magnetic data.

The complementary nature of the two methods ensured a more complete understanding of the subsurface architecture, supporting the hypothesis that Al-Dyabat may host a more extensive necropolis than previously known. The resemblance of the identified anomalies to typical Ptolemaic tomb designs, and the potential identification of high-status burials, highlight the site’s historical significance.

As one of the first geophysical studies conducted at this recently recognized site, the findings offer a reliable basis for prioritizing excavation areas while minimizing site disturbance. This approach aligns with modern archaeological practices that balance discovery with conservation.

Importantly, the methodologies and insights gained from this study hold broader global relevance. The successful integration of magnetic and GPR techniques at Al-Dyabat can serve as a replicable model for archaeological exploration in other heritage-rich regions facing similar preservation challenges. By promoting non-destructive, cost-effective, and efficient exploration strategies, this research contributes to international efforts aimed at protecting and understanding cultural heritage sites under threat from urbanization, climate change, and illicit excavation.

Ultimately, the integration of geophysical surveys with traditional archaeology represents a forward-looking approach, balancing discovery with conservation principles that will guide future explorations in this historically significant region and beyond.

## Data Availability

The data that support the findings of this study are available from the corresponding author upon reasonable request.
