# Correction: Non-invasive geophysical imaging reveals Ptolemaic tombs at Al-Dyabat for global archaeological applications

**DOI:** 10.1038/s41598-025-31718-3

**Published:** 2025-12-11

**Authors:** Abdelbaset M. Abudeif, Mohammed A. Mohammed, Hossameldeen A. Gaber

**Affiliations:** Faculty of science, Geology department, Sohag University, Sohag, Egypt

Correction to: *Scientific Reports* 10.1038/s41598-025-23113-9, published online 04 November 2025

The original version of this Article contained errors in the title of the paper. As a result:

“Non-invasive geophysical imaging reveals ptolemaic tombs at al-dyabat for global archaeological applications.”

now reads:

“Non-invasive geophysical imaging reveals Ptolemaic tombs at Al-Dyabat for global archaeological applications.”

Additionally, the formatting of the references in parts of text was incorrect. As a result, the formatting was changed as follows:

1. “Many authors used integration of geophysical methods to detect many archaeological features 2^18^.”

now reads:

“Many authors used integration of geophysical methods to detect many archaeological features^2–18^.”

2. “Its effectiveness has been well documented in numerous studies across Egypt^12,16,17^19^29^.”

now reads:

“Its effectiveness has been well documented in numerous studies across Egypt^12,16,17,19–29^.”

3. “Numerous geological investigations have been carried out to examine the lithostratigraphy of the sedimentary sequences in the Sohag region 39^44^.”

now reads:

“Numerous geological investigations have been carried out to examine the lithostratigraphy of the sedimentary sequences in the Sohag region^39–44^.”

4. “3D Euler Deconvolution (ED): Utilizes both vertical and horizontal derivatives to estimate source boundaries and depth^52^60^62^”

now reads:

“3D Euler Deconvolution (ED): Utilizes both vertical and horizontal derivatives to estimate source boundaries and depth^52,60–62^”

The original Article has been corrected.

